# Mapping the membrane orientation of auxin homeostasis regulators PIN5 and PIN8 in *Arabidopsis thaliana* root cells reveals their divergent topology

**DOI:** 10.1186/s13007-024-01182-7

**Published:** 2024-06-02

**Authors:** Yewubnesh Wendimu Seifu, Vendula Pukyšová, Nikola Rýdza, Veronika Bilanovičová, Marta Zwiewka, Marek Sedláček, Tomasz Nodzyński

**Affiliations:** 1 Mendel Centre for Plant Genomics and Proteomics, Central European Institute of Technology (CEITEC), Masaryk University, Kamenice 5, Brno, CZ-625 00 Czech Republic; 2 National Centre for Biomolecular Research, Faculty of Science, Masaryk University, Kamenice 5, Brno, CZ-625 00 Czech Republic

**Keywords:** *Arabidopsis thaliana*, Auxin homeostasis and transport, Digitonin PM-permeabilizing protocols, Endoplasmic reticulum, Immunocytochemistry, Membrane topology determination methods, PIN auxin efflux carriers

## Abstract

PIN proteins establish the auxin concentration gradient, which coordinates plant growth. PIN1-4 and 7 localized at the plasma membrane (PM) and facilitate polar auxin transport while the endoplasmic reticulum (ER) localized PIN5 and PIN8 maintain the intracellular auxin homeostasis. Although an antagonistic activity of PIN5 and PIN8 proteins in regulating the intracellular auxin homeostasis and other developmental events have been reported, the membrane topology of these proteins, which might be a basis for their antagonistic function, is poorly understood. In this study we optimized digitonin based PM-permeabilizing protocols coupled with immunocytochemistry labeling to map the membrane topology of PIN5 and PIN8 in *Arabidopsis thaliana* root cells. Our results indicate that, except for the similarities in the orientation of the N-terminus, PIN5 and PIN8 have an opposite orientation of the central hydrophilic loop and the C-terminus, as well as an unequal number of transmembrane domains (TMDs). PIN8 has ten TMDs with groups of five alpha-helices separated by the central hydrophilic loop (HL) residing in the ER lumen, and its N- and C-terminals are positioned in the cytoplasm. However, the topology of PIN5 comprises nine TMDs. Its N-terminal end and the central HL face the cytoplasm while its C-terminus resides in the ER lumen. Overall, this study shows that PIN5 and PIN8 proteins have a divergent membrane topology while introducing a toolkit of methods for studying membrane topology of integral proteins including those localized at the ER membrane.

**Supplementary Information:**

The online version contains supplementary material available at 10.1186/s13007-024-01182-7.

## Introduction

Auxin regulates plant growth and development through its concentration gradient, which is established by the activity of various auxin transporter proteins including auxin influx carrier AUX/LAX family and the auxin efflux PIN-FORMED (PIN) proteins [1]. The PIN-FORMED (PIN) proteins are a family of integral membrane proteins found in almost all land plants [2]. *Arabidopsis thaliana* genome encodes eight PINs which are classified into the plasma membrane (PM) localized PINs (PIN1, 2, 3, 4 and 7), the endoplasmic reticulum (ER) localized PINs (PIN5, and 8), and the dual PM- and ER-localized PIN6 [3–10]. The topology of PIN proteins is composed of trans-membrane domains (TMDs) separated by a central hydrophilic loop. The structure of the TMDs is highly conserved while the hydrophilic loop is varied in size and amino acid composition [9, 10]. The PM PINs have a longer central hydrophilic loop, the ER PIN5 and PIN8 have a shorter loop [3, 11], and PIN6 has an intermediate sized loop [12]. The central hydrophilic loop (HL) of the PM PINs localizes in the cytoplasm [8], and contains various motives, such as phosphorylation sites which modulate the polar PM distribution of the PINs [9, 13, 14]. The subcellular polarity of the PM PINs is essential to mediate the directional cell-to-cell auxin transport. Conversely, the ER-localized PIN5 and PIN8 do not have phosphorylation sites, and their role is to mediate the intracellular auxin homeostasis [3, 5, 10].

Studies have shown that PIN5 and PIN8 proteins have antagonistic activities in regulating the intracellular auxin homeostasis and various developmental aspects [5, 7, 11]. PIN5 enhances 1-naphthaleneacetic acid (NAA) accumulation in BY-2 tobacco cells, in contrast to PIN8 [11]. The authors also demonstrated that PIN5 promotes root hair growth while PIN8 inhibits it. A transport assay utilizing protoplasts isolated from a PIN5 overexpressing line revealed a decreased indole-3-acetic acid (IAA) export activity, whereas *pin5* knock-out mutants exhibited a higher level of free auxin. Therefore, it was proposed that PIN5 conveys auxin from the cytoplasm into the ER lumen [3]. In contrast, an analogue study using PIN8 overexpressing and *pin8* knock-out lines showed an increased and a decreased auxin export activity respectively, indicating that, antagonistically to PIN5, PIN8 may transport auxin from the ER lumen into the cytoplasm [5]. Data-driven modelling also shows that PIN5 protein mediates auxin flux into the ER lumen as opposed to PIN8 protein, which may export auxin out of the ER lumen [15]. In addition, the PIN5 overexpressing line showed a higher accumulation of IAA conjugates and a lower level of free IAA [3]. However, the PIN8 overexpressing plants exhibit a higher level of free IAA and a decreased accumulation of its conjugates [5]. Furthermore, the opposing activities of these proteins have been demonstrated in the analysis of root hair growth [11], lateral root development [16], hypocotyl growth [5], leaf venation pattern [7], and vein formation [17].

Although the opposite developmental roles of the ER-localized PIN5 and PIN8 and their countering influence on the intracellular auxin homeostasis have been reported, the mechanistic understanding why these proteins oppose each other remains unclear [5]. Furthermore, their native membrane topology has not been experimentally verified yet. We have previously published topology determination methods optimized for plant cells and PM-localized proteins [8]. In those protocols, the PM localized proteins fused with GFP reporter and subjected to apoplastic acidification or alkalization was used as a setup, and the resulting fluorescence change enabled to determine the topology of the long canonical PIN auxin efflux carriers. However, the short non-canonical PIN proteins localize at the ER membrane [3, 5, 9, 10], and an experimental verification of the membrane topology of the ER PINs is challenging due to lack of well-established protocols in plants which enable to determine the ins and outs orientation of their structure while the proteins are in their native membrane. Therefore, in this study, we optimized protocols which enable mapping the membrane topology of the ER PINs by preferentially permeabilizing the PM while the ER membrane remains intact. We implemented the protocol to study the membrane topology of the ER-localized PIN5 and PIN8 proteins expressed in *Arabidopsis thaliana* root cells. We mapped their main topological features such as the sub-cellular orientation of their central hydrophilic loop, and the N- and C-terminal ends. Our data show that, except for the similarities in the orientation of the N-terminus, PIN5 and PIN8 have opposite subcellular orientations of the central hydrophilic loop and the C-terminal end. The divergent topological arrangement of these proteins might provide structural ques explaining their antagonistic activities and points at the differences between these two intracellular auxin homeostasis regulators and the long PM-localized PINs [9].

## Materials and methods

### Plant material and growth conditions

The previously published *Arabidopsis thaliana* transgenic lines: PIN2::PIN8-GFP, PIN2::PIN5-GFP(-HL), and C1 (PIN5 chimeric protein which contains a GFP fused PIN2-HL inserted into the PIN5-HL) [18], 35S::PIN8-GFP(-HL) [5], AUX1::AUX1-YFP [19], PIN2::PIN2-GFP [20], PIN2::PIN1-GFP3 [21], SKU5::SKU5-GFP [22], 35S::HDEL-RFP [23], 35S::WER-GFP [24], and the T-DNA mutants *pin8-1* [5] and *pin5-5* [3] were utilized. The PIN5-GFP and PIN8-GFP fusions in the *pin5-5* or *pin8-1* background were obtained through genetic crosses. Homozygous lines were screened by T-DNA genotyping.

The seeds were sterilized with chlorine gas, plated on Murashige and Skoog medium (1% agar and 1% sucrose) and stratified at 4 ^o^C for 48 h in the dark. Seedlings were grown vertically at 21 ^o^C under 16 h : 8 h (light : dark) photoperiod.

### DNA constructs

To fuse the green fluorescent protein (GFP) at the N-terminus of PINs, the reporter sequence, without a stop codon, was PCR amplified and connected to PIN’s start codon using the*EcoRI* (New England Biolabs) restriction enzyme. For PIN-GFP1 constructs, the GFP was inserted into PIN genomic DNA in between 45 and 46 (in PIN5) or 30 and 31 (in PIN8)amino acids, using*Xbal* and*EcoRI* restriction enzymes. These PIN-GFP fusions were individually cloned into the Gateway entry vector pDONR™221 (Thermo Fishers Scientific). The 2.16 kb PIN2 promoter was cloned into the pENTR TOPO-TA vector (Thermo Fishers Scientific). The PIN2 promoter and each of the PIN-GFP entry clones were recombined intothe destination vector pH7m24GW_3 [25] by performing gateway LR reaction.

To prepare the PIN C-terminus GFP fusions, the PIN genomic DNA without a stop codon (-6 to 1889 bp for PIN5 and − 6 to 1776 bp for PIN8) was cloned into the pDONR™221 vector. Next, these entry clones were individually recombined with the PIN2 promoter and pDONR™ P2r-P3 (GFP entry clone), and the resulting expression clones were recombined into the destination vector pK7m34GW [25]. Transgenic *Arabidopsis thaliana* plants (Columbia ecotype) were generated by performing a floral dip using the *Agrobacterium tumefaciens* (strain GV3101). List of primers used in the study is available in the supplementary information (Table S1).

### The primary root length measurements

To verify the activity of the above described PIN-GFP fusions, we observed the primary root phenotype in the PIN-GFP expressing *Arabidopsis thaliana* transgenic lines. The seedlings were grown vertically on standard MS + media for 6 to 8 days. The seedlings were scanned, and the primary root length was measured using the ImageJ software (https://imagej.nih.gov/ij/). The quantified data were analyzed using R-Studio (Version 1.4.1717).

### Immunocytochemistry

To map the membrane topology of PIN5 and PIN8, we modified the whole-mount in situ immunodetection from the previously described protocol [8, 26, 27]. In the modified protocol, we utilized digitonin which were implemented in animal cell to permeate the plasma membrane without perforating the ER membrane [28]. We optimized the concentration of digitonin (10–40 µM) to permeate the plasma membrane while the ER membrane remains intact in *Arabidopsis thaliana* root cells. We used the anti-GFP or anti-BiP primary antibodies (1:500, Sigma-Aldrich) raised in Mouse, and the anti-Mouse secondary antibody conjugated with CY3 (1:600, Sigma-Aldrich).

### Acidification or alkalization treatment

To verify the topology of PM PINs, we performed selective acidification or alkalization of the apoplast using the protocol described previously [8]. To map the topology of ER PINs in reference to the ER membrane, we optimized the GFP quenching protocol which was established for studying topology of the plasma membrane localized auxin transporter proteins [8, 19]. The cytosolic pH was lowered by HCl (pH 5.0) co-treatment with digitonin (10 µM) for 30 min.

### Confocal microscopy and fluorescent signal analysis

We observed the GFP, RFP, anti-GFP, and anti-BiP fluorescent signals using the confocal microscopy (Carl Zeiss LSM 700 or 780 system). All images were taken with a 40X water objective. The fluorescent signal was quantified using the Fiji software (https://fiji.sc). Figures were assembled in Inkscape (inkscape.org).

## Results

To determine the membrane topology of PIN5 and PIN8 proteins, in terms of the sub-cellular orientation of their central hydrophilic loop (HL) and the two terminals, we utilized various PIN-GFP fusion proteins expressed in *Arabidopsis thaliana* root cells. The previously published 35S::PIN8-GFP [5], and PIN2::PIN5-GFP constructs [18], which contain the GFP tag in their central hydrophilic loop (HL) allowed us to map the sub-cellular orientation of the HL. To map the sub-cellular position of the two terminals of both proteins, we generated the PIN2::PIN5-GFP and PIN2::PIN8-GFP fusions, which contain the GFP tag at their N- or C-terminal ends.

### The PIN5 and PIN8 N- and C-terminal GFP fusions localize at the ER

To check whether the N- or C-terminal GFP fusions of PIN5 and PIN8 localize at the endoplasmic reticulum (ER), we performed a Brefeldin A (BFA) treatment, and immuno-localization utilizing anti-BiP antibody, whereby the BiP is an ER-localized chaperone [29]. The plasma membrane localized proteins are internalized into the BFA compartment while the ER localized proteins do not form the BFA aggregation [30], and the canonical PM PINs also form BFA compartment [11, 18]. If these PIN5-GFP and PIN8-GFP fusions localize at the ER, the GFP signal should co-localize with the anti-BiP immuno-staining, and should not show BFA induced intracellular aggregations. A clear co-localization of the PIN5-GFP and PIN8-GFP fusions with the BiP ER marker (Fig. S1A, B), and the absence of BFA aggregations (Fig. S1C), indicate that these PIN-GFP fusions, similar to the previously described PIN5 [3] and PIN8 proteins [5], localize to the ER.

### PIN5 and PIN8 exhibit an antagonistic effect on primary root growth

**Fig. 1:**
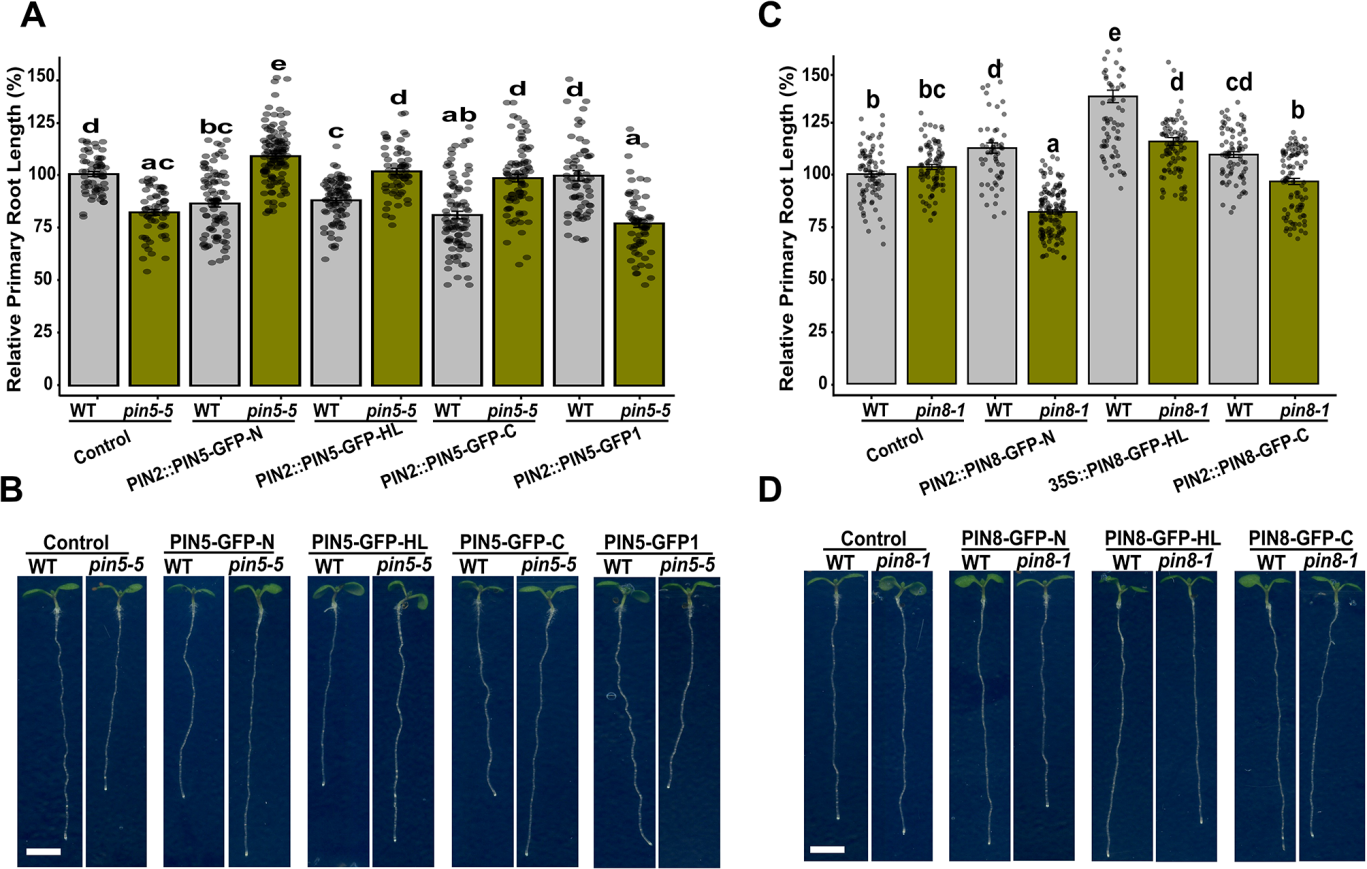
PIN5 and PIN8 exhibit antagonistic effects on primary root elongation. (**A**, **C**) Quantified primary root length from 6 days old seedlings. (**B**, **D**) Root phenotype of PIN5-GFP and PIN8-GFP fusions in the WT, and *pin5-5* or *pin8-1* mutant background. The designation, N, C, or HL in the PIN-GFP label indicates the position of GFP tag at the N- or C-terminal of PINs or in the central hydrophilic loop, respectively. Error bars represent standard error of mean. The dots in the bars indicate individual data points obtained from at least 60 seedlings per line. The letter labels above the bar indicate significant differences (*P* < 0.05) by General Linear Model, gaussian family and identity link function followed by Tukey’s multiple comparison test

To assess the functionality of our newly generated constructs, we investigated root phenotype of the PIN5-GFP and PIN8-GFP fusions both in the WT and *pin5-5* [3], or *pin8-1* [5] mutants background, respectively. First, we analyzed the activity of the PIN5-GFP fusions. The *pin5-5* mutant exhibits a shorter hypocotyl and primary root [3]. Complementing the *pin5-5* mutant with the ER localized PIN2::PIN5-GFP-N and PIN2::PIN5-GFP-C fusions or with the PM localized PIN2::PIN5-GFP-HL restored primary root (Fig. 1A, B) and hypocotyl elongation (Fig. S2C, D) of the mutant to the WT level, indicating that these PIN5-GFP fusions are functionally active.

In comparison to the WT control, the expression of the PIN5-GFP fusions in the WT background inhibit the primary root growth (Fig. 1A, B), showing that the presence of the additional WT copy of the PIN5 gene in the ER membrane may be enhance the activity of the protein, which may result in the inhibition of primary root elongation.

The similarity in the primary root phenotype of the plasma membrane and the ER localized PIN5-GFP fusions, may also show that the plasma membrane localization of the PIN5-GFP line [18], may not change the activity of the protein. However, the PIN2::PIN5-GFP1 fusion, which contains the GFP in the second TM helix did not complement both the primary root (Fig. 1A, B) and the hypocotyl of the *pin5-5* mutant (Fig. S2C, D), indicating that the insertion of the GFP in the helix of this construct may interfere with the activity of the protein due to the disruption of its structure, and therefore the PIN5-GFP1 construct seems non-functional.

Next, we tested the activity of PIN8-GFP fusions by analyzing primary root elongation and lateral root density. The primary root phenotype of *pin8-1* mutant resembles the WT seedlings (Fig. 1C, D). In comparison to the WT control, the expression of the PIN2::PIN8-GFP N- and C-terminal GFP fusions and the previously published 35S::PIN8-GFP-HL overexpression line [5], in the WT background enhanced the primary root elongation. However, in the *pin8-1* mutant background, all PIN8-GFP fusions grew shorter root than their phenotype in the WT background (Fig. 1C, D). Furthermore, in agreement with the previous study [16], we observed that the *pin8-1* mutant is defective in lateral root density, and all PIN8-GFP fusions restored lateral root density in the *pin8-1* mutant (Fig. S2A, B). These results show that the PIN8-GFP fusions are functional and the GFP tag did not interfere with their activity.

### Immunolocalization revealed the N-terminal end and the central hydrophilic loop of PIN5 reside in the cytoplasm while its C-terminus faces the ER lumen

The functionality of the PIN-GFP fusions mentioned above allowed us to use them as valid tools for mapping their membrane topology. First, we mapped the topology of PIN5. We performed immunodetection using IGEPAL (2%) to permeate all cellular membranes [8, 27] or digitonin (40 µM) to preferentially permeabilize the PM [26, 28]. Permeabilization with IGEPAL enables the labelling of epitopes situated both in the cytoplasm and in the ER lumen [27]. However, permeating the plasma membrane with digitonin enables us to label the epitopes residing in the cytosol but not inside the ER. Simultaneous immunodetection using the two protocols enables to differentiate between an epitope positioned in the cytoplasm or in the ER lumen.

**Fig. 2:**
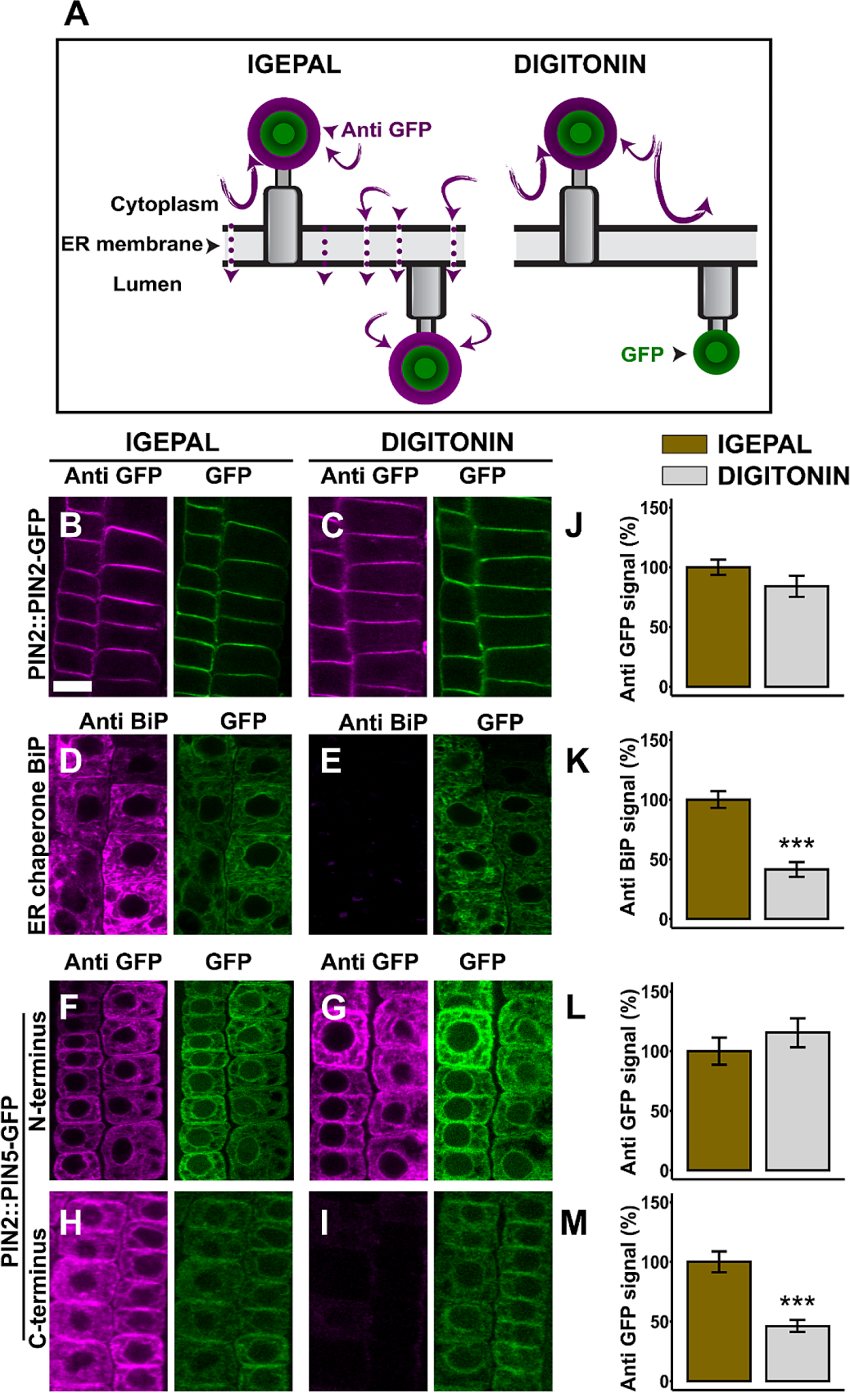
PIN5 has its N-terminus in the cytoplasm and its C-terminus in the ER lumen. (**A**) Cartoons showing the principle of ER membrane permeable versus non-permeable immunocytochemistry. IGEPAL permeates all cellular membranes and allows antibodies to detect epitopes positioned in the cytoplasm and the ER lumen. Digitonin permeates the PM alone but not the ER membrane and enables to label the cytoplasmic epitope alone, but not the luminal epitope. (**B**–**I**) Representative epidermal root cell pictures for immunolocalization. The designations anti-GFP or GFP refer to the immuno-staining and the native GFP fluorescence signal respectively while the anti-BiP refers to the BiP immuno-staining signal. To label the ER chaperone BiP, as a luminal control, PIN2::PIN5-GFP transgenic line was used. (**J**–**M**) Quantified immunofluorescence signal intensity. The IGEPAL (control) immunofluorescence is plotted as 100%. The asterisks indicate significant differences in comparison to PM permeabilization with IGEPAL (*** *P* < 0.001, Student’s t-test). The significantly lower signal after digitonin treatment compared to IGEPAL indicates the ER lumen orientation of protein moiety. The error bars represent SEM from the total number of seedlings analyzed from three biological experiments (*n* > 15 per experiment). Scale bar = 10 μm

In a control experiment carried out to label the plasma membrane localized PIN2-GFP, which has the GFP insertion in its cytoplasmic central hydrophilic loop, the antibody detected the GFP both in the IGEPAL and digitonin permeated cells (Fig. 2B, C). In addition, the quantified anti-GFP signal after permeabilizing the cells with either IGEPAL or digitonin was similar (Fig. 2J), indicating that digitonin permeates the plasma membrane as effectively as IGEPAL. To test whether the concentration of digitonin used in this experiment permeabilized the plasma membrane, but not the ER membrane, we performed similar experiment using the BiP protein, a HSP70 chaperone located in the ER lumen [29]. Permeabilization with IGEPAL allowed the immunodetection of the chaperone (Fig. 2D, K). However, after permeating the cell by digitonin, the anti-BiP antibody did not label the protein (Fig. 2E, K), showing that the antibody did not access the luminal BiP because the ER membrane remained intact.

Consistently with the PIN2-GFP immunodetection, the GFP tag at the N-terminal end of PIN5 was clearly detected after permeabilization with IGEPAL or digitonin (Fig. 2F, G, L), indicating that the N-terminal end of the protein is oriented cytoplasmically. In addition, we performed a similar experiment to label the PIN5-GFP-1 insert. This construct contains the GFP insertion in between the 45th and 46th amino acid sequence, and this GFP position is in the second TM helix, according to AlphaFold PIN5 model. The anti-GFP labelled the GFP-1 moiety only in the IGEPAL permeated cells (Fig. S3A, B, E). Although the PIN5-GFP1 may not represent the native topology of the PIN5 protein, the detection of the GFP-1 moiety only after PM permeabilization indicates that the GFP1, which may be embedded in the second TM helix at the luminal side of the lipid bilayer, is protected by the ER membrane. This indicates that the digitonin did not perforate the ER membrane and the protocol is good enough to differentiate between the cytoplasmic and the luminal epitopes. The C-terminal end of PIN5-GFP was detected in the IGEPAL permeated cells alone, but not in the digitonin permeated cells (Fig. 2H, I, M), showing that the carboxy terminus of PIN5 may be oriented on the luminal side of the ER membrane.

Although PIN5 localizes at the ER [3], (Fig. S1), the plasma membrane localization of this protein [18] provided the possibility to determine the topology of its hydrophilic loop (HL), in which the GFP was inserted. We performed immunocytochemistry with and without permeating the plasma membrane. In this study, the immunolocalization technique in which the plasma membrane was permeated by IGEPAL after tissue fixation with paraformaldehyde (PFA) [27] and glutaraldehyde (GA) was designated as plasma membrane permeable protocol. This technique allows to label epitopes localized at the apoplast and in the cytoplasm. Excluding membrane permeabilization detergents from the protocol enables to maintain intact plasma membrane. This technique is limited to label extracellularly positioned epitopes alone and referred to as membrane non-permeable protocol [8].

**Fig. 3:**
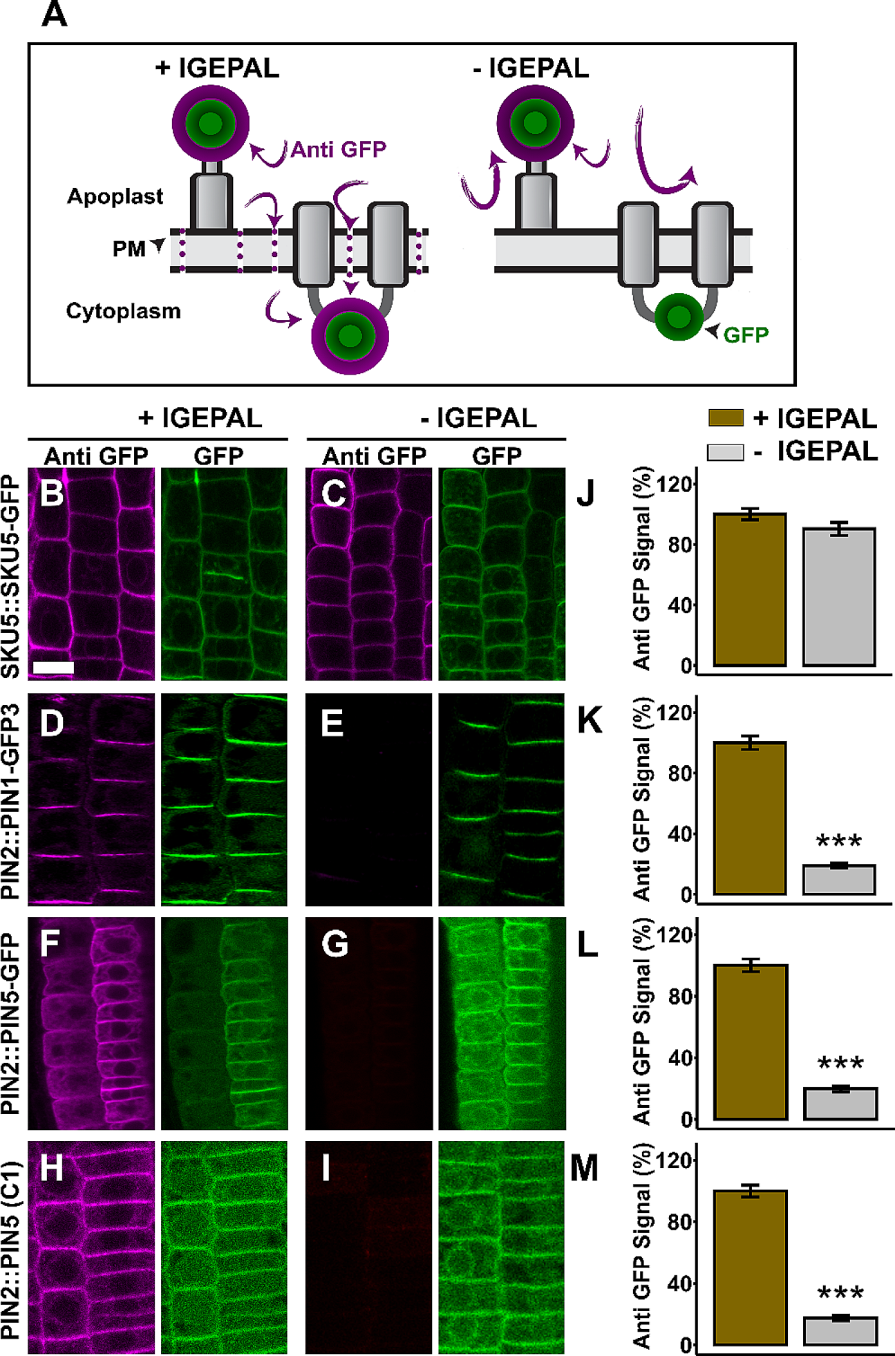
The central hydrophilic loop of PIN5 is found in the cytoplasm. (**A**) Cartoon showing the principle of PM permeable versus non-permeable immunostaining. PM permeable protocol using IGEPAL allows to label both apoplastic and cytoplasmic epitopes. The PM non-permeable protocol in the absence of IGEPAL limited the antibody to detect only the apoplastic epitope. (**B**-**I**) Representative root pictures for PM permeable and PM non-permeable immunolocalization. The plasma membrane permeable (+ IGEPAL) immuno-fluorescence is plotted as 100%. The asterisks indicate significant differences in comparison to PM permeabilization with IGEPAL (*** *P* < 0.001, Student’s t-test). The presence or absence of anti-GFP signal in the plasma membrane non-permeable condition (- IGEPAL) indicates the apoplastic or cytoplasmic orientation of the epitopes respectively. (**J**-**M**) Quantified immunofluorescence signal intensity. Error bars represent SEM from the total number of seedlings analyzed from three biological experiments (*n* > 18 per experiment)

In a control experiment carried out using the extracellularly positioned SKU5-GFP, the epitope was clearly labelled both in the membrane permeable and non-permeable immuno detections (Fig. 3B, C, J). However, the anti-GFP antibody labelled the cytoplasmic PIN1-GFP3 (Fig. 3D, E, K) only upon membrane permeabilization with IGEPAL. Moreover, in an analogical experiment conducted with PIN5 (C1-GFP), PIN5 chimeric protein which contains GFP tagged PIN2-HL in the PIN5-HL [18], the antibodies label the C1-GFP moiety in the membrane permeable immunolocalization alone (Fig. 3H, I, M). Therefore, if the PIN1-GFP3 and C1-GFP epitopes cannot be detected without membrane penetration, it indicates that the plasma membrane permeable versus non-permeable immunodetection protocol was effective enough to distinguish between the apoplastic and the cytoplasmic epitopes. Similarly, the PIN5-GFP fusion, which do not contain the PIN2 hydrophilic loop, was clearly labelled under the membrane permeable immunolocalization alone, while detection of the protein was abolished in the membrane non-permeable protocol (Fig. 3F, G, L), indicating that the HL of PIN5 localizes in the cytoplasm.

### The N- and C-terminal ends of PIN8 are oriented towards the cytoplasm while its hydrophilic loop faces the ER lumen

**Fig. 4:**
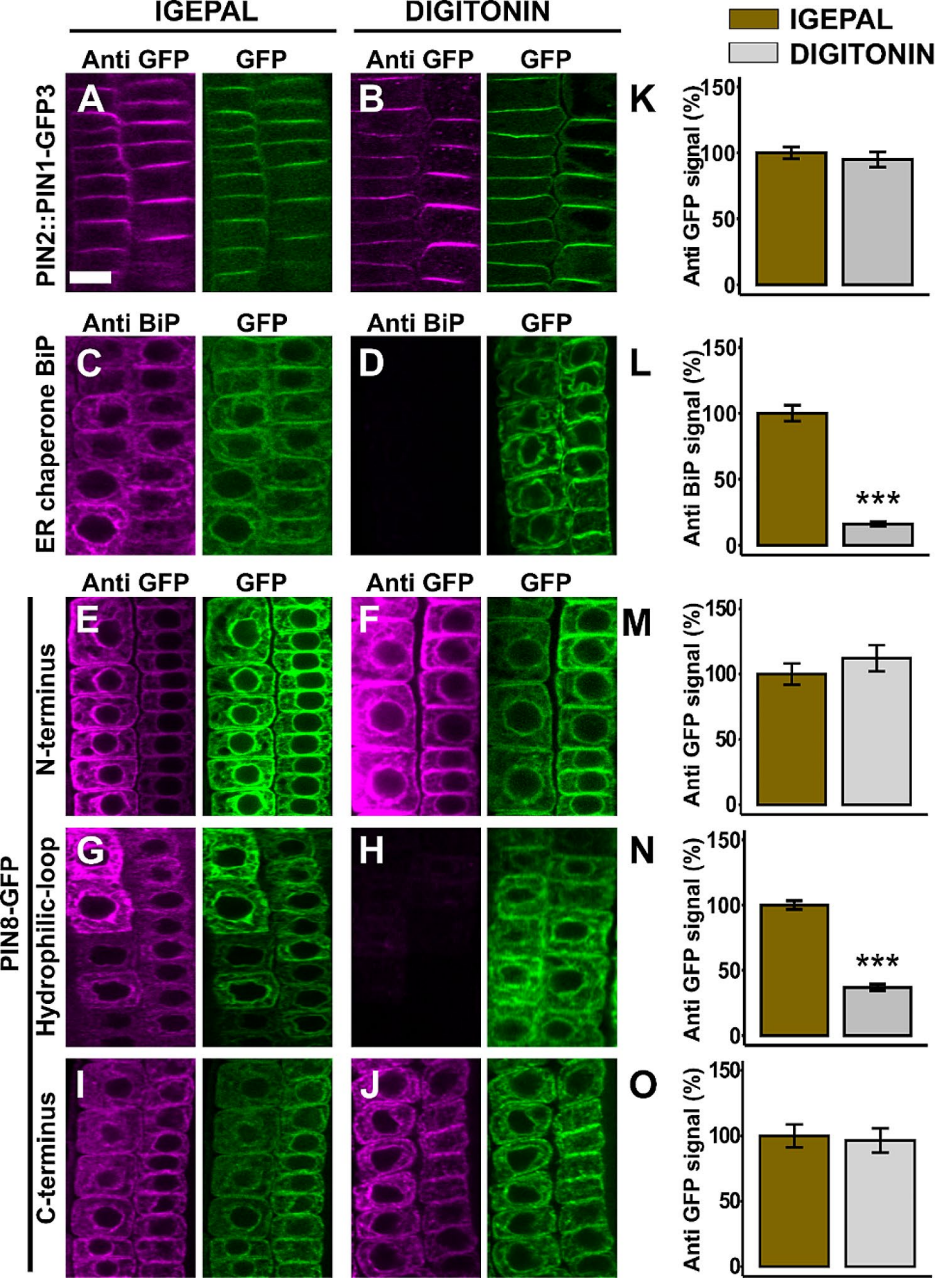
PIN8 has its N - and C - terminus in the cytoplasm while its central hydrophilic loop localizes in the ER lumen. (**A**-**J**) Representative root pictures after anti-GFP immunolocalization. The anti-GFP or GFP labels in the figures indicate the anti-GFP or GFP fluorescence signal while anti BiP is the BiP immunodetected fluorescent signal. To label the ER chaperon BiP as a luminal control, PIN2::PIN8-GFP line was used. (**K**-**O**) Quantified immunofluorescence signal intensity. The immunofluorescence after the IGEPAL permeabilization (control) is plotted as 100%. The asterisks indicate significant differences in comparison to permeabilization with IGEPAL (*** *P* < 0.001, Student’s t-test). The significantly lower immuno-fluorescence signal after digitonin treatment compared to the IGEPAL indicates the ER-luminal orientation of the reporter. Error bars represent SEM from total number of seedlings analyzed from three biological experiments (*n* > 15 per experiment). Scale bar = 10 μm

Next, we mapped the topology of PIN8, using immunolocalization technique adapted for studying membrane topology of the ER localized PIN5 protein described in the above. In these sets of experiments, we used the cytoplasmic PIN1-GFP3 [8], as a control construct with a known topology. As expected, like the PIN2-GFP (Fig. 2B, C, J), the GFP3 moiety was clearly detected after permeating the cell with either IGEPAL or digitonin (Fig. 4A, B, K). However, the ER-luminal BiP immuno detection revealed almost no signal in digitonin-permeated cells in comparison to the IGEPAL treatment (Fig. 4C, D, L). This consistently demonstrated that the ER lumen is not accessible to antibodies when digitonin alone is used in the immuno-localization protocol. In the case of the PIN8-GFP fusions, like the PIN1-GFP3, the N-terminal (Fig. 4E, F) and the C-terminal (Fig. 4I, J) were clearly labelled with the anti-GFP antibody both in the IGEPAL and digitonin-permeated cells. There was statistically no significant difference between the quantified anti-GFP signal corresponding to IGEPAL and digitonin treatment both in the PIN1-GFP3 (Fig. 4K) and PIN8-GFP N- and C-terminus (Fig. 4M, O). These results indicate that the two terminal ends of PIN8 protein orient on the cytoplasmic side of the ER membrane. In contrast, the GFP immuno-detection of PIN8-GFP1, in which we inserted the GFP in between the first two helices of the N-terminal domain, was significantly lower in digitonin permeated cells, in comparison to the IGEPAL permeabilized cells (Fig. S3C, D, F). This result shows that PIN8 contains its first minor loop in the ER lumen, and this conclusion supports the cytoplasmic orientation of its N-terminus. Furthermore, to determine the orientation of PIN8 HL, we took advantage of the previously generated ER-localized 35S::PIN8-GFP [5]. The immuno detection of the central HL of PIN8 showed a markedly weaker labelling in digitonin permeated cells in contrast to permeabilization with IGEPAL (Fig. 4G, H, N). This shows that PIN8 contains its central HL in the ER lumen, which is oriented to the opposite position of its terminus.

In addition, we further tested the subcellular orientation of PIN8 HL using the plasma membrane localized PIN2::PIN8-GFP line [18]. Since the subcellular orientation of PIN8-HL in reference to the ER membrane is in the lumen, the orientation of the HL of this protein in reference to the plasma membrane should be in the apoplast. To confirm this, first, we performed a protease protection assay on alive root cells using trypsin. If the HL of PIN8 is oriented outside the cell, the GFP in the HL of PIN8 should be degraded upon trypsin treatment. The previously described PIN8-GFP transgenic line [18], has a dual PM and the ER membrane localization. But there is a substantial portion of PIN8 on the PM that should be accessible to trypsin. Following trypsin treatment, we isolated the membrane fraction of proteins from Arabidopsis root cells expressing this construct and performed western blot to immuno-detect the presence or absence of GFP. We utilized PIN2-GFP and SKU5-GFP as cytoplasmic and apoplastic controls respectively. The PIN2-GFP and the PIN5-GFP (C1) proteins responded to the trypsin treatment in a comparable manner. However, in comparison to the PIN2-GFP, the apoplastic SKU5-GFP was significantly degraded after trypsin treatment (Fig. S4). This result shows that the cytoplasmic GFP moiety is highly protected by the PM from being digested by the trypsin while the extracellularly positioned GFP is highly degraded in response to the enzymatic digestion. Similar to the SKU5-GFP, the fraction of PIN8-GFP disappeared almost entirely in response to the trypsin treatment (Fig. S4). This result indicates that the position of PIN8 HL in relation to the plasma membrane is in the apoplast, and this agrees with the luminal orientation of the HL when the protein localizes at the ER membrane (Fig. 4).

### Selective acidification of the cytoplasm or the apoplast largely corroborates the membrane topology of PIN5 and PIN8

**Fig. 5:**
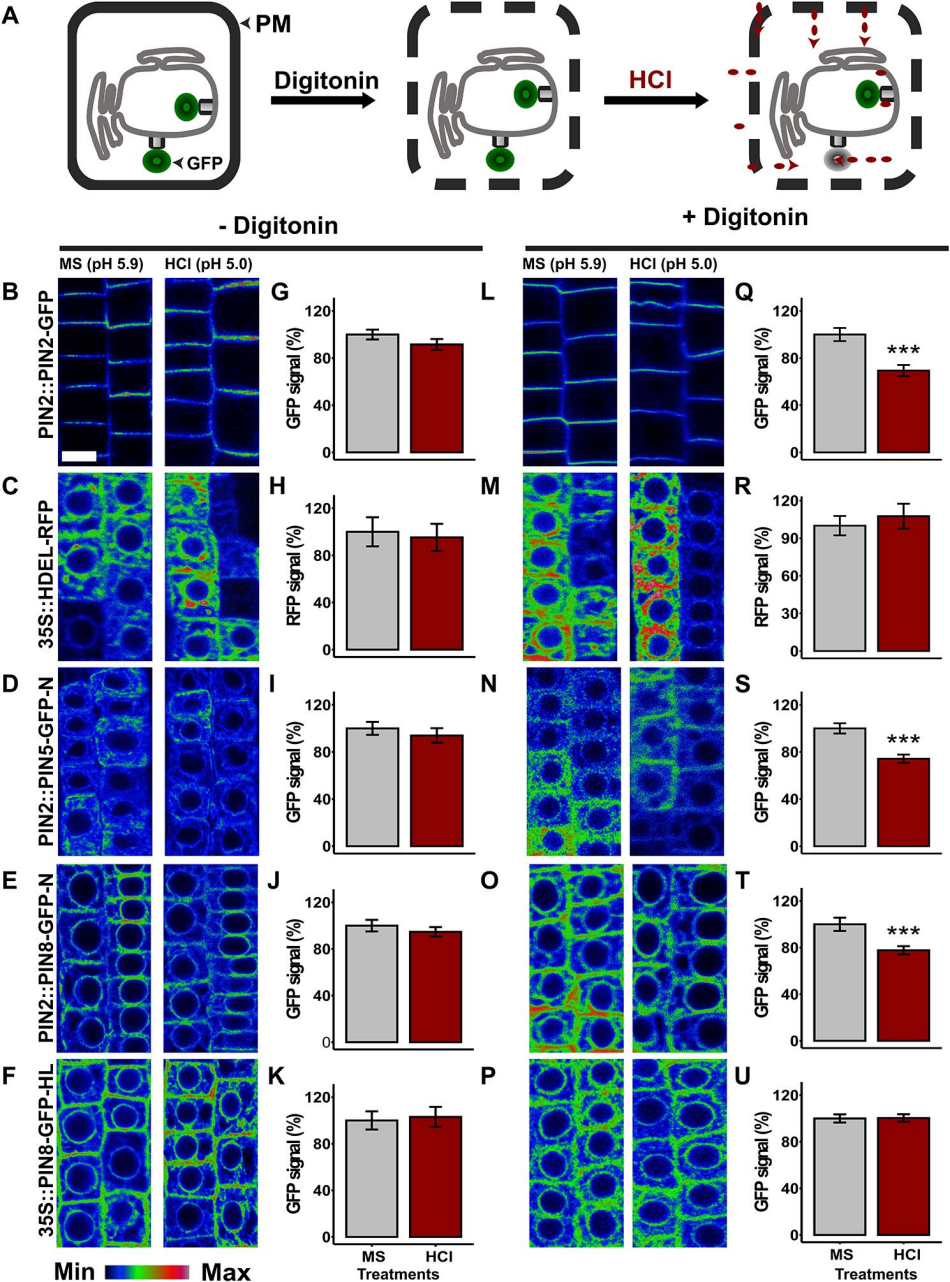
In the absence of digitonin, the PM non-permeable HCl does not quench the intracellular fluorescence while permeating the PM with digitonin allows quenching the cytoplasmic fluorescent reporters. (**A**) Cartoon showing the principle of intracellular GFP quenching. Acidification of the cytosol after permeating the PM with digitonin decreases the fluorescence of the cytoplasmic GFP reporters while the ER-luminal ones are not affected. (**B**-**F**) Color-coded root pictures from a live cell imaging showing that acidification of the apoplast did not affect the intracellular fluorescent reporter signal. (**G**-**K**) Quantified fluorescent signal from seedlings subjected to acidification of the apoplast. (**L**-**P**) Pictures from root cells exposed to acidification of the cytoplasm. (**Q**-**U**) Quantified fluorescent signal after acidification of the cytoplasm. The GFP or RFP (mCherry) signal in the MS (control) treatment is plotted as 100%. The asterisks indicate significant differences in comparison to the control MS treatment (*** *P* < 0.001, Student’s t-test). Error bars represent SEM from the total number of seedlings analyzed from three biological experiments (*n* > 15 per single experiment). Scale bar = 10 μm

To verify the results described above we utilized GFP as a pH-sensitive probe [31], and adopted a fluorescent protein quenching assay [8, 19], for studying the topology of ER-localized PIN-GFP proteins in a live root cell. To evaluate the applicability of the method, we first assessed the effect of the HCl (pH = 5.0) on the fluorescent reporter signal. The PIN2-GFP in which the fluorescent moiety faces the cytoplasm, the HDEL-RFP which resides in the ER lumen, and PIN5-GFP and PIN8-GFP fusion proteins which localize at the ER were incubated (30 min) in liquid Murashige and Skoog medium (MS, pH = 5.9 (control)) or the MS medium titrated with hydrochloric acid (HCl, pH = 5.0). The HCl should not affect the GFP and RFP fluorescence of the intracellular chimeric proteins because the PM is not permeable to the protons generated after HCl dissociation in the titrated MS medium [8]. Both in the PIN2-GFP and HDEL-RFP, the acid treatment did not affect the fluorescent signal (Fig. 5B, C, G, H). Similarly, the HCl did not quench PIN5-GFP and PIN8-GFP fusion proteins (Fig. 5D-F, I-K). These results indicate that acidification of the apoplast does not affect the intracellular reporters. Therefore, to utilize the HCl-mediated fluorescent quenching for studying the topology of a protein in reference to the ER membrane, the PM needs to be permeated.

To permeate the PM in live root cells, we optimized the minimum concentration of digitonin (10 µM), which should not affect the PIN-GFP fluorescent signal. To assess the effect of digitonin on the GFP signal, the cytoplasmic PIN2-GFP, and the ER localized PIN8-GFP and PIN5-GFP transgenic lines were incubated in liquid MS medium with and without digitonin. The GFP signal in all constructs subjected to digitonin treatment was similar to their respective control MS medium without digitonin (Fig. S5). These results show that the concentration of digitonin used to permeate the membrane in live root cells did not affect the GFP fluorescent signal.

To permeate the PM and to acidify the cytosol, six days old seedlings expressing PIN-GFP fusions were subjected to digitonin co-treatment with either MS (control) or HCl. If digitonin permeates the PM in a live root cell, but not the ER membrane, the fluorescent signal of GFP situated in the cytoplasm should be decreased in response to the acid treatment while the one which is enclosed in the ER lumen should remain unaffected. After permeating the PM with digitonin, the acid treatment quenched the cytoplasmic PIN2-GFP and significantly decreased the fluorescent signal in comparison to the respective MS control treatment (Fig. 5L, Q). This result indicates that digitonin perforates the PM which allows the HCl to diffuse into the cytoplasm and quenches the GFP moiety. To check whether digitonin permeates only the PM, but not the ER membrane, an analogical experiment was conducted by using HDEL-RFP, in which the RFP moiety is enclosed in the ER lumen. If the digitonin permeates the ER membrane, the HDEL-RFP signal should be diminished in response to the acid treatment. However, after the digitonin and acid co-treatment, the RFP signal remained as high as the fluorescent signal in the control treatment (Fig. 5M, R). To check whether the HDEL-RFP stability in this experiment was due to the RFP resistance to the lower pH or because it was protected by the ER membrane, we performed a similar experiment by using MS medium titrated with propionic acid (pH = 5.0). Since the propionic acid is membrane permeable, it should quench the luminal fluorescence. As expected, the HDEL-RFP signal was significantly degraded after treatment with propionic acid (Fig. S6). These results collectively suggest that the insensitivity of the RFP to the HCl, although the PM was permeated with the digitonin, is due to its enclosure in the ER lumen, but not because of its resistance to the acidic pH.

After permeating the PM with digitonin, similar to the observation in the PIN2-GFP, the fluorescent signal both in the PIN5 and PIN8 N-terminal GFP tag was significantly quenched by the HCl (Fig. 5N, O, S, T), confirming that the N-termini of both proteins are in the cytoplasm. Subjecting the GFP moiety to the acidic pH titrated with HCl does not cause protein degradation [8]. The GFP signal loss at the acidic pH is related to the pH-induced secondary structural distortion of the tag [31]. However, PIN8-GFP1 lines, which contain the GFP in the small loop extending from the first TM-helices at the opposite side of the N-terminus, remained stable after the digitonin and acid co-treatment (Fig. S3 H, J). These results indicate that the first minor loop in the N-terminal domain of the PIN8 is in the ER lumen. Similarly, the GFP moiety in the PIN8 hydrophilic loop was not affected by the acidification of the cytosol (Fig. 5P, U), showing that the HL of the protein is enclosed in the ER lumen. These results agree with the immunocytochemistry findings.

Furthermore, to check the sub-cellular localization of PIN5 HL, we took advantage of the ectopic PM localization of PIN2::PIN5-GFP line, which contains the GFP insertion in its central HL [18]. This transgenic line was subjected to either acid or base treatment using MS (control, pH = 5.9) titrated with HCl (pH = 5.0) or KOH (pH = 8.0). Both HCl and KOH are PM non-permeable [8]. If the reporter moiety is extracellularly localized, the acidic pH decreases the fluorescent signal while the alkaline treatment enhances it [8]. The glycosyl phosphatidylinositol-anchored SKU5-GFP, in which the fluorescent reporter is positioned at the exterior of the cell [22] and the PIN2-GFP, which contains the GFP tagged HL located in the cytoplasm [8], were used as control constructs with a known topology.

**Fig. 6:**
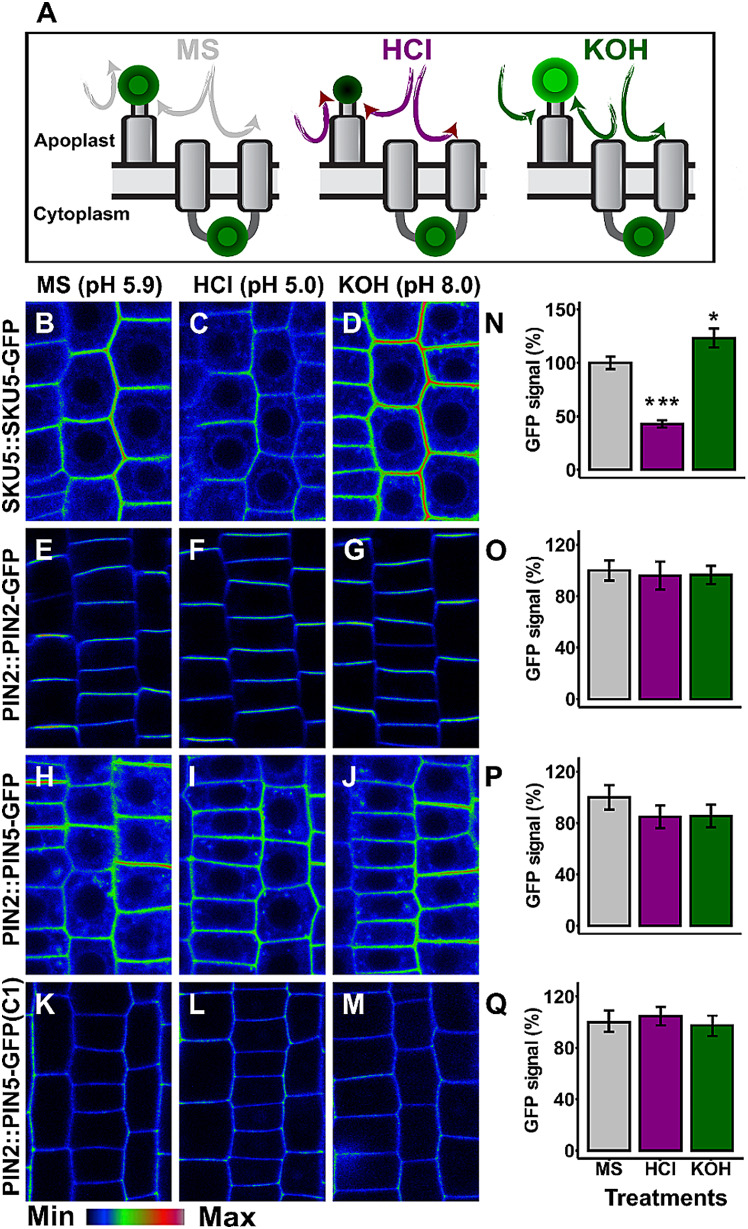
GFP quenching reveals the cytoplasmic position of the PIN5 hydrophilic loop. Six days old seedlings were subjected to standard Murashige Skoog Medium (pH = 5.9, control) titrated with either HCl (pH = 5.0) or KOH (pH = 8.0) for 30 min. (**A**) Cartoon showing the principle of the extracellular GFP quenching. (**B**-**M**) Root images are shown in signal intensity color code to better visualize the GFP intensity changes after the acidic or alkaline treatment. (N-Q) Quantified GFP signal intensity. The control treatment (MS) per each construct was plotted as 100%. The asterisks indicate significant differences in comparison to the control MS treatment (*** *P* < 0.001, *P < 0.05, Student’s t-test). The error bars represent SEM from the total number of seedlings obtained from three biological experiments (*n* > 18 per single experiment)

The acid treatment significantly decreased the fluorescent signal in the apoplastic SKU5-GFP (Fig. 6B, C, N) in comparison to the respective control medium (MS, pH = 5.9), while a non-significant change was observed in the PIN2-GFP (Fig. 6E, F, O), PIN5-GFP (Fig. 6H, I, P) and C1 (PIN5-PIN2-HL-GFP) (Fig. 6K, L, Q). Furthermore, the alkalization treatment (KOH, pH = 8.0) enhanced the GFP signal only in the SKU5-GFP (Fig. 6B, D, N), but not in the other transgenic lines (Fig. 6G, J, M, O, P, Q). These results support the above mentioned immunolocalization findings (Fig. 3F, G, H, I, L, M), which show that the HL of PIN5, like that of PIN2, is oriented in the cytoplasm.

**Fig. 7:**
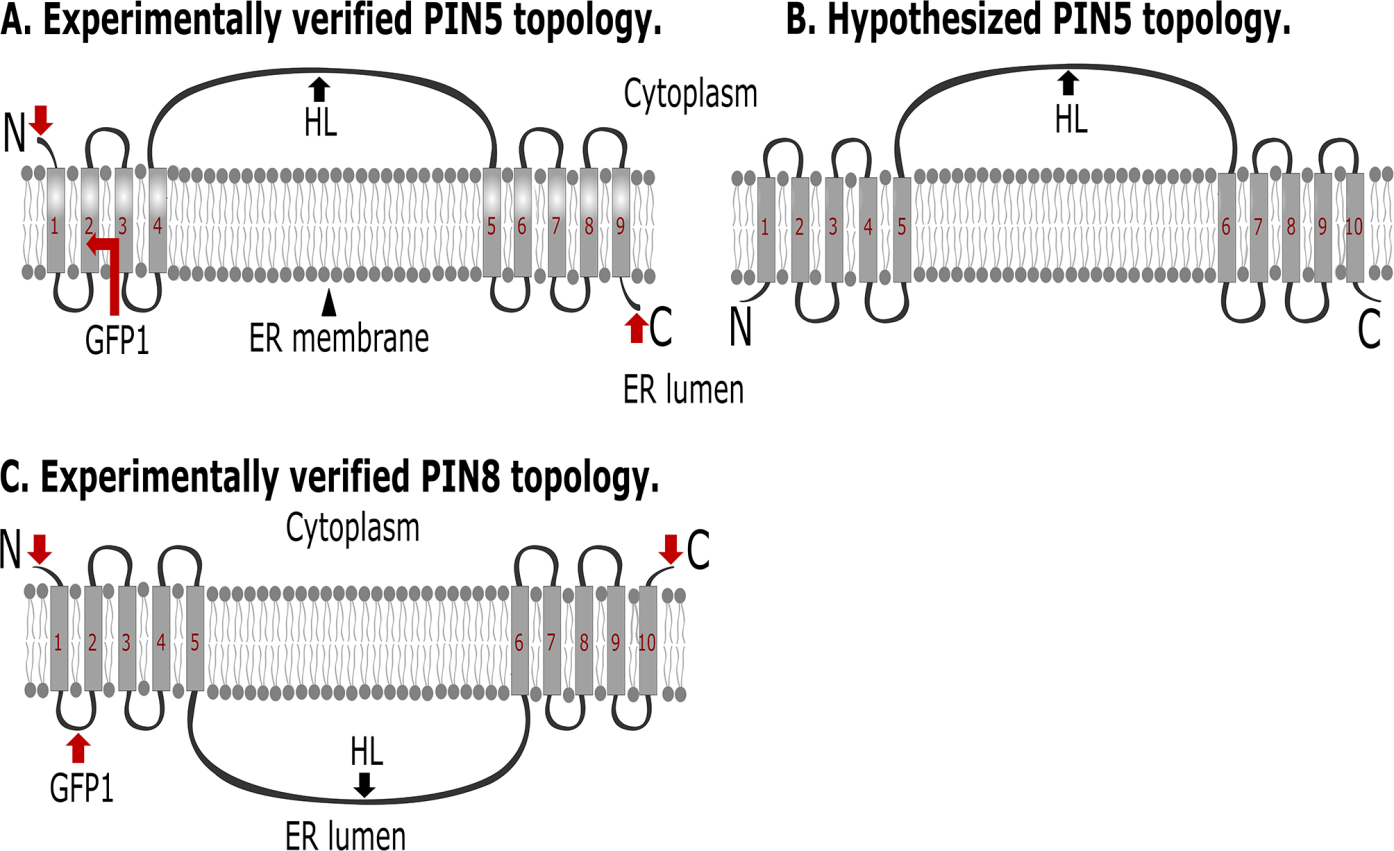
Model of membrane topology of PIN5 and PIN8 proteins. The designation ’’N’’, ‘’C’’, and ‘’HL’’ represents the N- or C-terminal end of the protein, and its central hydrophilic loop respectively. The red arrows indicate the position of GFP insertion in the PIN5-GFP and PIN8-GFP fusions

Next, we mapped the full structure of PIN5 and PIN8 proteins by combining our experimental data with the 3D structure of the proteins. AlphaFold provides an almost accurate prediction for the 3D structure of different transmembrane proteins, including PINs [32]. However, its prediction for the central HL of PIN5 and PIN8 is very low (pLDDT < 50) (Fig. S7). The authors declared that regions with a very low pLDDT might be unstructured in isolation. The PIN8 AlphaFold model shows similar orientation of its N- and C-terminal end (Fig. 7), and this agrees with our experimental data (Fig. 4), and the published structure [33].

Unlike the AlphaFold model, our topology experimental data for PIN5 structure indicate an opposite orientation of its terminal ends (Fig. 2), hinting that the structure of PIN5 might be different from that of PIN8. *Arabidopsis thaliana* PINs amino acid sequence alignment also revealed that PIN5 helices contain some residues which are distinct from the other PIN’s conserved amino acids (Fig. S8). Based on our experimentally verified topology, and the two versions of *Arabidopsis thaliana* PIN5 gene AlphaFold models, which show ten or eight TM helices (Fig. S7), we provided an alternative (hypothesized) structure of the protein (Fig. 7).

## Discussion

Although the mutually opposing role of PIN5 and PIN8 proteins in regulating the intracellular auxin homeostasis [5], and various developmental aspects have been reported [5, 7, 11, 17], how the two proteins which localize at the same intracellular compartment (endoplasmic reticulum) antagonize each other remains unclear [5]. In this study, we determined the membrane topology of PIN5 and PIN8, in terms of the sub-cellular orientation of their N- and C-terminal ends, and the central hydrophilic loop. Our results showed that, despite the similarities in the orientation of the N-terminus, PIN5 and PIN8 have divergent membrane topology. Therefore, their topological differences could show their mutually opposing activity or at least highlight their differences that are still debated.

The cytoplasmic and the ER luminal localization of the N- and C- terminus of PIN5, respectively (Fig. 2), implies that the protein’s topology is composed of an odd number of transmembrane domains. In addition, the cytoplasmic position of its central hydrophilic loop (Figs. 3 and 6) indicates that PIN5 has four and five transmembrane helices in its N- and C-terminal domain, respectively (Fig. 7A). The PIN5 experimental structural data do not agree with its 3D AlphaFold model [32]. The discrepancy between the PIN5 experimentally verified structure and its AlphaFold model is not easy to explain. However, it is true that the *Arabidopsis thaliana* PIN5 gene AlphaFold model is not verified, as two models of the protein which show ten or eight helices exist (Fig. S7). On the other hand, although the PIN5-N-terminal GFP fusion complements the *pin5-5* mutant and remains functional (Fig. 1A, B, S2C, D), if the GFP tag might be exposed to the cytoplasm regardless of the membrane orientation of the PIN5 protein, the GFP moiety can be detected after permeating the plasma membrane with digitonin. From the methodological point of view, the protocol is still valid and good enough to differentiate between the luminal and the cytoplasmic epitopes. Therefore, if the cytoplasmic detection of the PIN5-N terminal fusion may not represent the topology of PIN5, it is possible that the N-terminal end of the protein, like its C-terminal end (Fig. 2), may also reside in the ER lumen. Based on this hypothesis, we proposed an alternative structure of the protein, which contains ten TM helices whereby its cytoplasmic central HL (Figs. 3 and 6) is positioned after the fifth helix, and both terminal ends reside in the ER lumen (Fig. 7B).

The cytoplasmic position of both termini of PIN8 (Fig. 4) indicates that this protein has an even number of transmembrane domains. This finding agrees with the topology model obtained from UniProt [34], and AlphaFold database [32, 35], which shows ten-TMDs of the protein. The experimentally verified structure of PIN8, using cryo-electron microscopy also showed ten TMDs of PIN8 structure [33]. In contrast to our findings, in terms of the ins and outs orientation of the two termini and the central HL of the protein, the previous study reported that the HL of PIN8 localizes in the cytoplasm while the two termini possess non-cytoplasmic position [33]. However, it is worth noting that in this study the PIN8 protein is expressed heterologously, purified, and reconstituted into peptidiscs, small patches of artificial membranes. This process does not include the membrane topology preserving machinery, which facilitates insertion, integration and folding of the protein within the biological lipid bilayer. We believe that in such artificial systems orientation of the reconstituted proteins might be changed from the innate one. In this study, we mapped the membrane topology of ER PINs in the cells of the organism where they are expressed naturally and in their native membranes. The inward and outward facing confirmational structure of the protein [33], may be caused by the presence of IAA or NPA. In our study we did not make either the IAA or NPA treatment, and it seems that we did not detect the confirmational changes in the structure of the protein. Hence, the plausible membrane topology of PIN8 may contain ten TMDs, which is separated into groups of five alpha-helices by the central hydrophilic loop residing in the ER lumen (Figs. 4G, H and N and 7C), or in the apoplast in reference to the plasma membrane (Fig. S4).

The cytoplasmic and the ER luminal localization of the central HL and C- terminus of PIN5, respectively (Figs. 2, 3 and 6), in opposite to the orientation of PIN8’s HL and C-terminus (Fig. 4), implies that these proteins have a divergent membrane topology. *Arabidopsis thaliana* PIN sequence alignment shows that some PIN5 residues differ from the other PIN’s conserved amino acids, both in the scaffold and transporter domains (Fig. S8) [33]. Furthermore, at the beginning of the sixth TM helices (near the end of the central HL), PIN5 retains unique “EKSFLEVMSL” residues. However, PIN6, PIN8, and all canonical PINs (PIN1–4, and 7) conserve “IVMMRLILTV”, “SVGTMKILLK,” and “SVMTRLILIM” residues respectively, and contain “IL” in common before the last two residues of the conserved amino acids (Fig. S8) [33]. We believe that multiple amino acid substitution in a protein sequence could affect the entire structure of the protein, and the structural differences between the PIN5 and PIN8 proteins might be related to the variations in their amino acid sequence composition. In addition, in the recent report of the PIN8 structure, PIN5 does not cluster together with PIN8, which is grouped closer to the canonical long PINs [33]. This result might also reflect the divergent structure of PIN5 and PIN8, and an independent structural evolution of noncanonical PINs [36].

The topology of polytopic membrane proteins is determined during their biogenesis at the ER membrane. At the ER, the signal recognition particle (SRP) recognizes the first TM helix as it emerges from the ribosome and targets the nascent helices to the ER membrane, where the Sect. 61 translocon facilitates their integration into the lipid bilayer [37]. Likewise, the membrane topology of PIN5 and PIN8 shows that the topogenesis of both proteins depend on signals encoded in the amino acid sequence that guide the integration of their first TM helices into the ER membrane. Once the protein is anchored in the ER membrane, its topological orientation is highly determined by the positive inside rule [38, 39]. Based on this paradigm, although additional factors can influence the overall topology, residues with net positive charge are preferentially oriented to the cytoplasm [37, 39].

In eukaryotic cells, the membrane potential across the ER membrane, which might regulate the positive inside rule is negligible and the origin of this rule is not clear [37]. However, the net negative charges around translocon and lipid headgroups might maintain the cytosolic retention of the more positive signals [37]. The charged residues in the Sec61p translocon sub-unit favoring the positive inside rule has been observed in yeast (*Saccharomyces cerevisiae*) [39], and the *sec61p* mutants are less efficient to maintain this rule [40]. It seems that the charge distribution of the ER PINs which vary from one another and also from the PM localized PINs [41], follow the positive inside principle. The highly negative residues in the PIN8’s central HL (Fig. S9), support its localization in the ER (Fig. 4G, H, N), or in the apoplast (Fig. S4), when examined in the PM localizing variant [18]. Congruently, the less negative HL of PIN5 (Fig. S9) localizes to the cytoplasm regardless of if we assay its topology at the ER or PM. That is consistent with the earlier findings that the overall topology of protein is preserved and does not flip flop in the lipid bilayer even without strong charge bias incorporated in the amino acid sequence [42].

The divergent membrane topology of PIN5 and PIN8 may reflect their opposing activity in mediating the intracellular auxin homeostasis and developmental events. The *pin5-5* mutant, which is defective in hypocotyl and primary root elongation, accumulates high level of free IAA in rosette leaves and root tips [3]. However, decreased level of free IAA and pronounced capacity to accumulate IAA amino acid conjugates following induction of PIN5 gene expression has been observed in BY-2 cells [3]. This indicates that the mechanism of the *pin5-5* mutant complementation with the PIN5-GFP fusions (Fig. 1A, B), might be by enhancing the IAA conjugation, which may limit free the IAA supply to the nucleus and prevent primary root inhibition. TIR1/AFB-Aux/IAA signaling pathway is needed for auxin-induced primary root growth inhibition [43]. However, the PIN5-GFP induced inhibition of primary root elongation in the WT seedlings expressing the PIN5-GFP fusions could be due to the presence of the additional WT copy of the PIN5 may promote auxin flux into the ER. Studies have proposed that PIN5 transports auxin into the ER lumen [3, 15], from where it could reach the nucleus [15].

In contrast to PIN5, overexpression of PIN8 increases and decreases the level of free IAA and its conjugates, respectively [5]. The shorter roots in the PIN8-GFP fusions expressed in the *pin8-1* mutants than the WT seedlings expressing the same construct (Fig. 1C, D), might be related to an elevated level of free IAA following expression of the protein. The *pin8* mutant is defective in lateral root density, and the PM localized AUX1 protein restored lateral root density in *pin8-1* mutant while PIN5 fail to rescue the mutant [16]. The AUX1 mediated mechanism of restoring lateral root in the *pin8-1* could be by enhancing the cytoplasmic free IAA though its IAA influx activity. These studies collectively indicate that, the PIN8 favours accumulation of cytoplasmic free IAA, maybe by fluxing IAA into the cytoplasm [5, 15] and by inhibiting IAA conjugation [5], through downregulation of auxin conjugating GRETCHEN HAGEN3 (GH3) gene [4]. In addition, the *pin8* mutant exhibited a reduced pollen transmission while *pin5* mutant showed a WT phenotype. However, the *pin5 pin8* double mutants combination rescued the *pin8* mutant pollen transmission defect to a WT level [5]. The countering activity of these proteins have been further demonstrated through data driven modeling [15], and in the analysis of leaf venation pattern [7], root hair growth [11], hypocotyl elongation [5], and vein formation [17].

In general, our study established a tool kit to determine membrane topology of ER localized integral proteins and showed the overall opposite structure of PIN5 and PIN8, which could advance the discussion on the features and evolution of these noncanonical PINs. To make straightforward functional comparisons between PIN5 and PIN8 proteins, and to unambiguously relate topology of these proteins with their activity or auxin transport direction across the ER membrane, detailed auxin transport assays will have to be conducted in plant cells or plant derived membranous organelles.

### Electronic supplementary material

Below is the link to the electronic supplementary material.

Supplementary Material 1

## Data Availability

The data that supports this study are included in this article and its supplementary material.
