# Supplementary material for: Mapping the membrane orientation of auxin homeostasis regulators PIN5 and PIN8 in Arabidopsis thaliana root cells reveals their divergent topology
Source: Plant Methods. 2024 Jun 2;20:84. doi: 10.1186/s13007-024-01182-7 (PMC11145782; doi:10.1186/s13007-024-01182-7)
Supplement: Supplementary file 1 — Supplementary Material 1 [file 13007_2024_1182_MOESM1_ESM.docx]

#


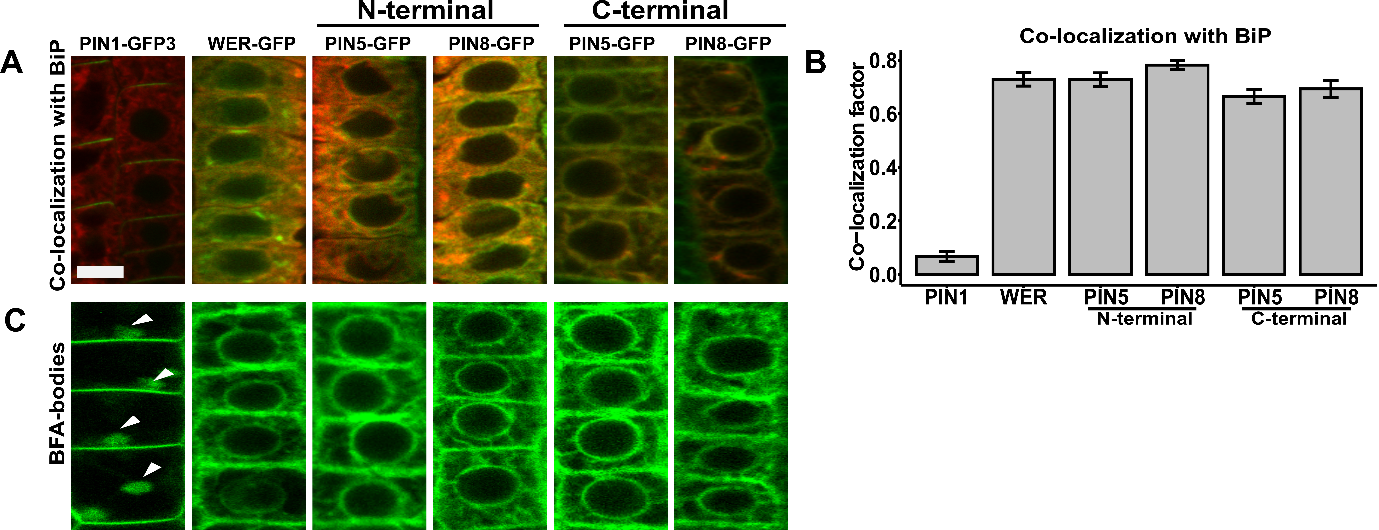


**Fig. S1.** PIN5 and PIN8 localize to the ER**.**

The N- and C-terminally GFP tagged PIN5 and PIN8 localize at the ER. The PIN-GFP fusions were expressed under PIN2 promoter in the *Arabidopsis thaliana* root cell. PIN1-GFP3 and WER-GFP were utilized as a control for the plasma membrane and endoplasmic reticulum localization respectively. (A-B) PIN5 and PIN8 co-localize with the ER chaperon BiP. The co-localization factor was determined using Zeiss software, where the co-localization factor close to 1 shows strong co-localization. (C) The BFA aggregation in the plasma membrane localized PIN1-GFP3 (indicated by the white arrows) and the absence of BFA aggregations both in PIN5 and PIN8 indicates the absence of these proteins in the PM derived endosomes. Five days old seedlings were incubated in BFA (25 µM) for one hour. For microscopy observation, 5 days old seedlings (n = 5) and at least 10 epidermal root cells per seedling were included in the analysis. The error bars represent the SEM. Scale bar, 10 µm.


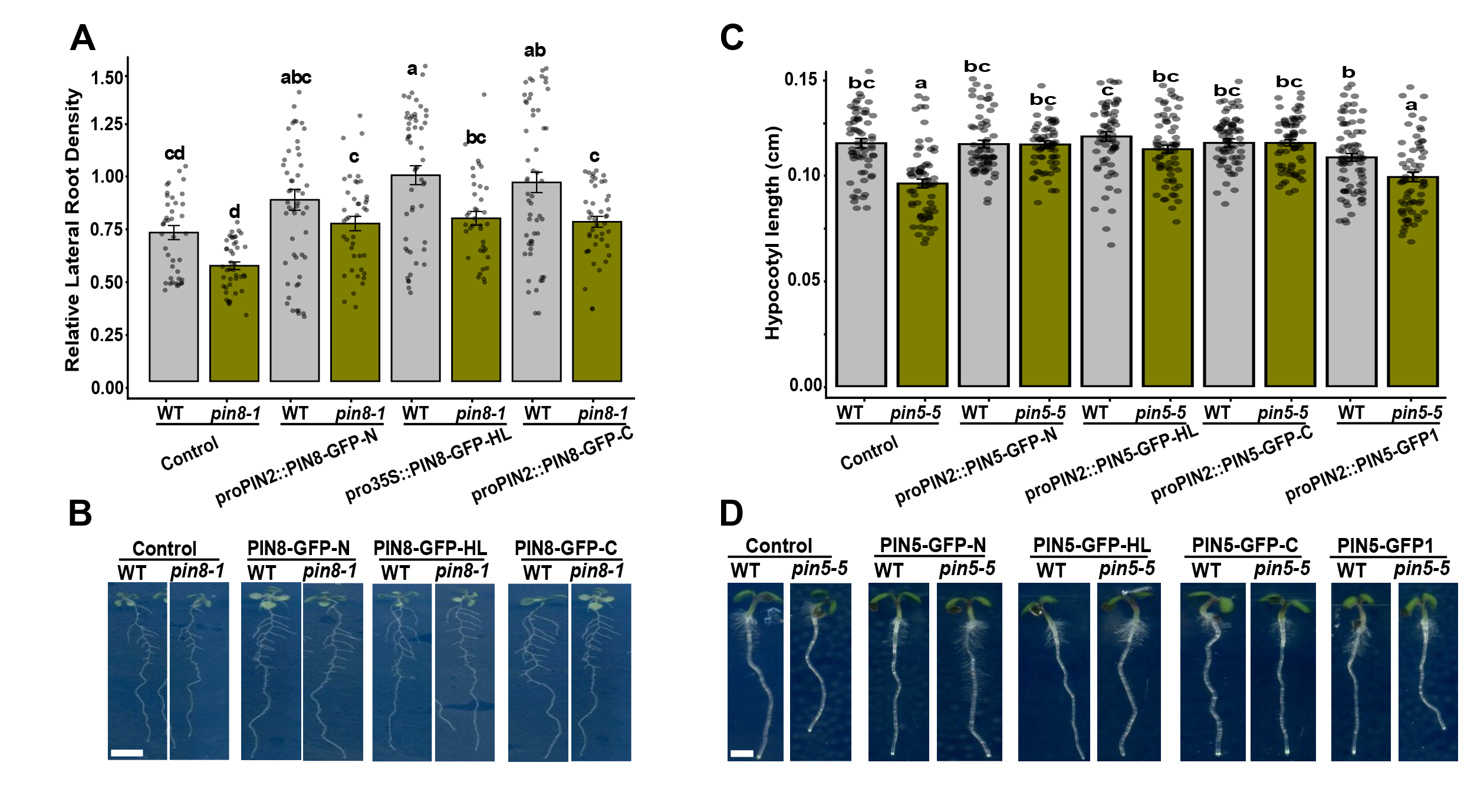


**Fig. S2.** PIN8-GFP and PIN5-GFP fusions rescued *pin8-1* and *pin5-5* mutants respectively. (A) Quantified relative lateral root density from 11 days old WT, *pin8-1* mutant, and PIN8-GFP fusions expressing seedlings. The dots in the bar indicates individual data points obtained from at least 38 seedlings per each line.

(B) Lateral root phenotype of PIN8-GFP fusions in the WT or *pin8-1* mutant background.

(C) Quantified hypocotyl length (the stem of the seedlings) from five days old WT, *pin5-5* mutant, and PIN5-GFP fusions expressing seedlings. The dots in the bars indicate individual data points obtained from at least 50 seedlings per each line.

(D) Seedlings to show PIN5 hypocotyl phenotype in the WT, *pin5-5* mutant, and PIN5-GFP fusions expressing lines.

Error bars represent standard error of mean. The letter labels above the bar indicate significant differences (*P* < 0.05) by General Linear Model, gaussian family and identity link function followed by Tukey’s multiple comparison test.


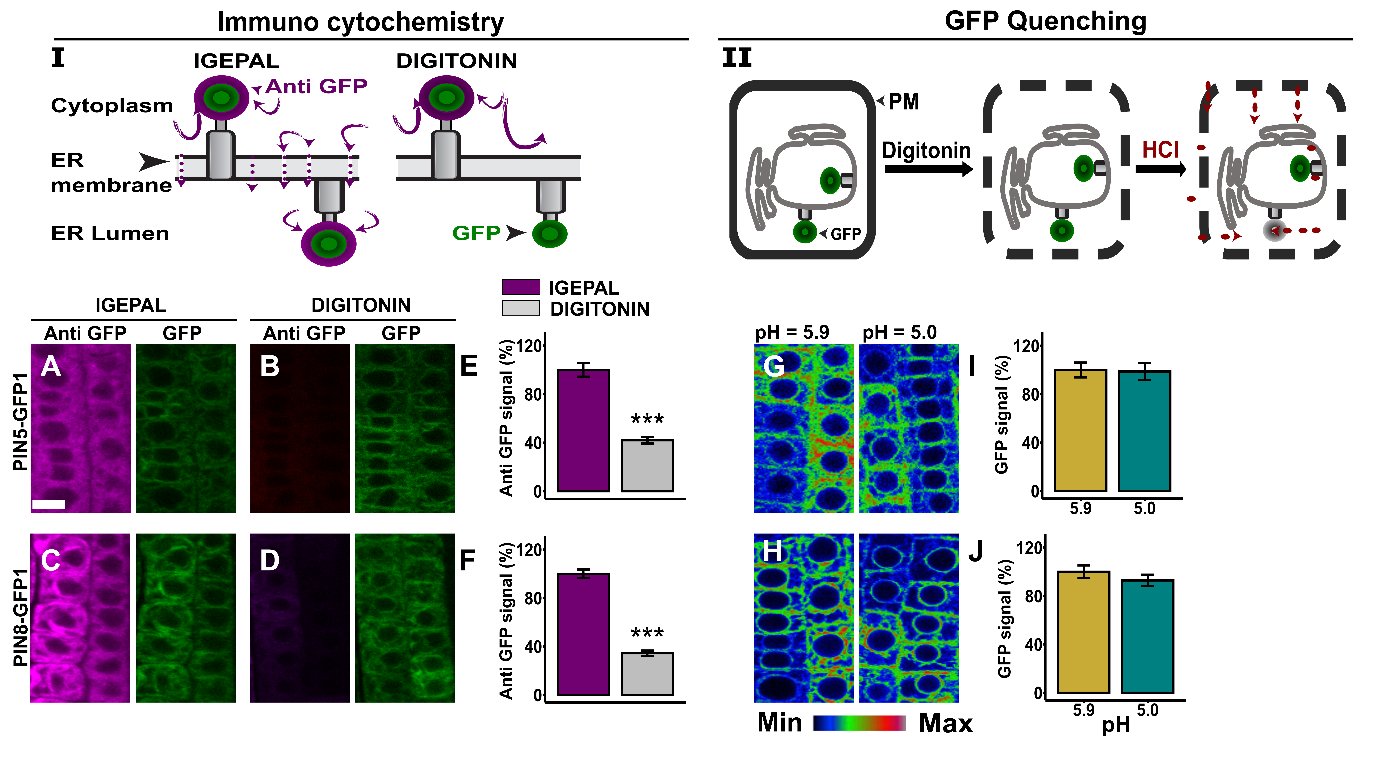
**Fig. S3.** Immuno-cytochemistry and GFP quenching of lines with reporter insertion in the GFP1 position. The GFP was inserted in the second TM helix in PIN5, and in the small loop located between the first and the second helices in PIN8. The cartoons presented in roman number I and II indicate the principle of immunocytochemistry and GFP quenching to map membrane topology of ER proteins. (I) In the principle of ER membrane permeable versus non-permeable immunocytochemistry, IGEPAL permeates all cellular membranes and allows antibodies to detect epitopes positioned in the cytoplasm and the ER lumen. Digitonin permeates the PM alone but not the ER membrane and enables to label the cytoplasmic epitope alone, but not the luminal epitope. (II) In the principle of intracellular GFP quenching. acidification of the cytosol after permeating the PM with digitonin decreases the fluorescence of the cytoplasmic GFP reporters while the ER-luminal ones are not affected.

(A-D) The immuno-detection of the GFP was abolished in digitonin permeated epidermal root cells. (E-F) The quantified anti GFP signal. The control treatment (IGEPAL) was plotted as 100%. The asterisks indicate significant differences in comparison to PM permeabilization using IGEPAL (*** *P* < 0.001, Student’s t-test). The error bars represent SEM from the total number of seedlings obtained from three biological experiments (n > 18 per single experiment). (G-H) Root epidermal cell images shown in signal intensity color code to better visualize the GFP intensity changes after the acidic or alkaline treatment. Six days old seedlings were co-treated by either digitonin and MS+ or digitonin and HCl for 30 minutes. (I-J) Quantified GFP fluorescent signal. Error bars represent SEM from the total number of seedlings analysed from three biological experiments (n > 15 per single experiment). Scale bar, 10 µm.


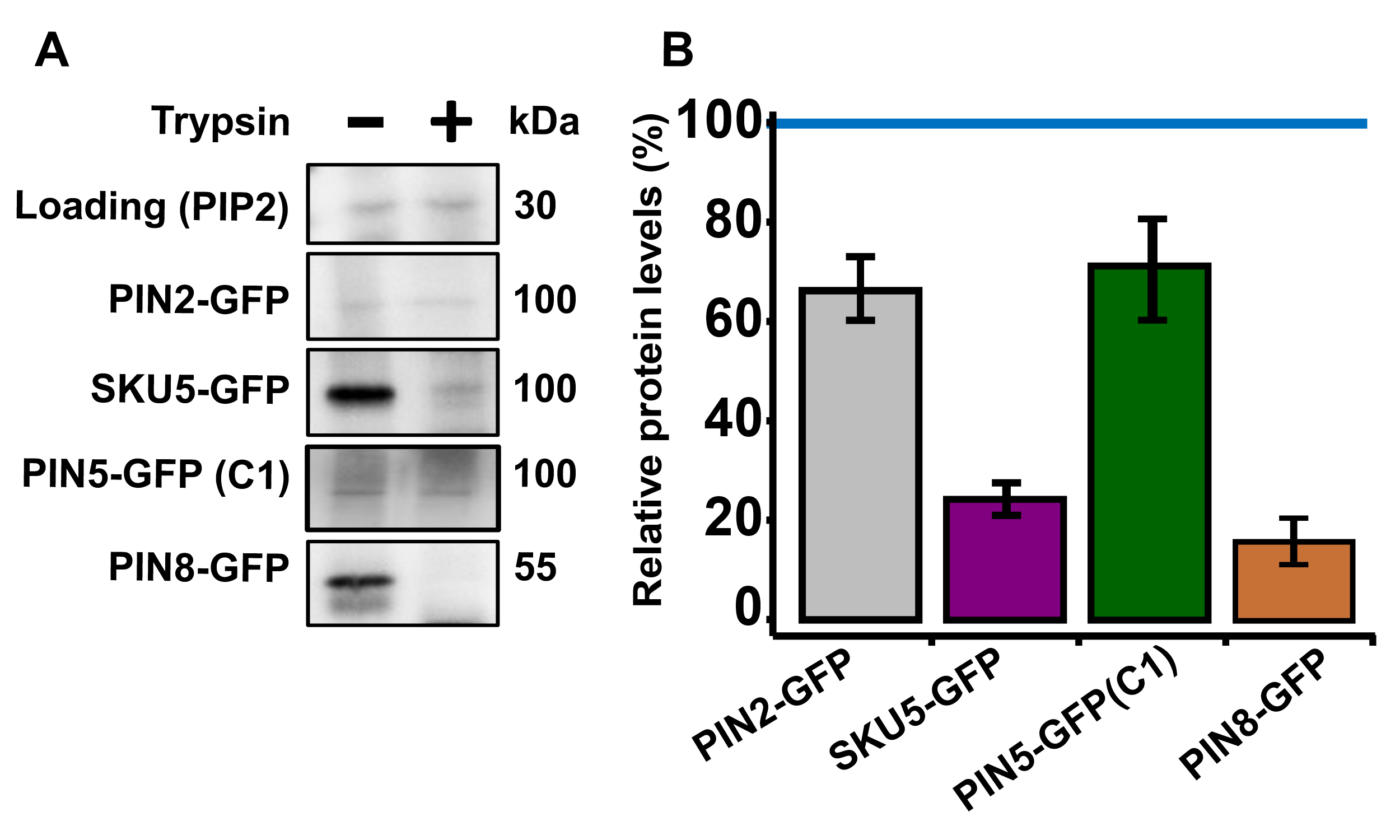


**Fig. S4.** Trypsin protection assay combined with western blot analysis indicating the apoplastic orientation of PIN8 HL. *Arabidopsis thaliana* transgenic lines expressing PIN2-GFP, SKU5-GFP, PIN5-GFP (C1), and PIN8-GFP were treated with trypsin (40 µM) for 20 minutes at 37 ^o^C. Afterwards, membrane fraction was isolated from the root cells and GFP was detected in western blots. (A) Western blots indicating presence and/or absence of GFP-tagged protein after mock or trypsin treatment. PIP2 represents the loading control. (B) Quantification of the relative protein levels from the western blot visualised protein bands. The blue line above the graph indicates the level of protein in the control (- trypsin) sample. Error bars represent standard error of mean of 3 biological repeats.  After the extracellular application of trypsin, the protein levels of SKU5-GFP and PIN8-GFP were highly decreased indicting apoplastic position of the GFP tag.


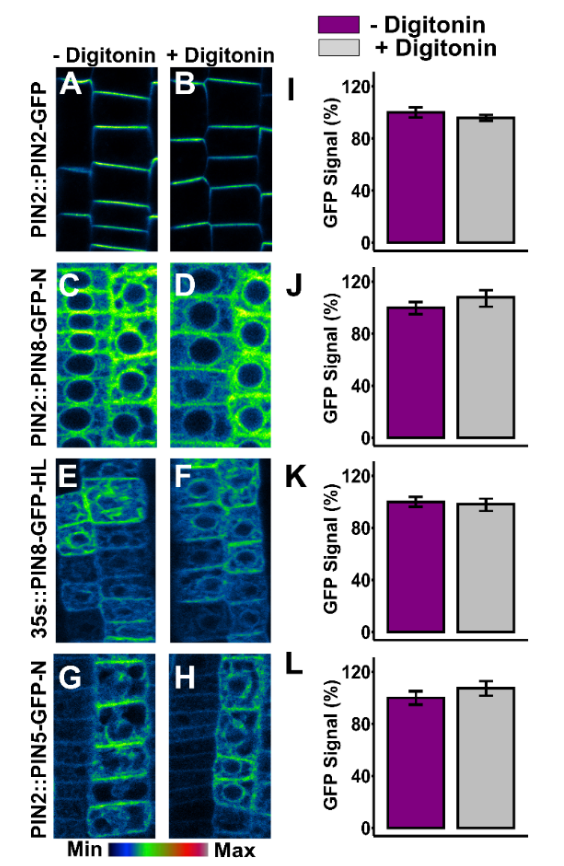


**Fig. S5.** Digitonin does not affect the green fluorescent protein signal.

(A-H) Epidermal root cell images shown in signal intensity color code to better visualize the GFP intensity changes before and after digitonin treatment. Six days old seedlings were treated by either MS+ (control) or MS+ with digitonin for 30 minutes. (I-L) Quantified GFP fluorescent signal. The error bars represent SEM from three independent biological experiments (n > 10 per experiment). Scale bar, 10 µm.


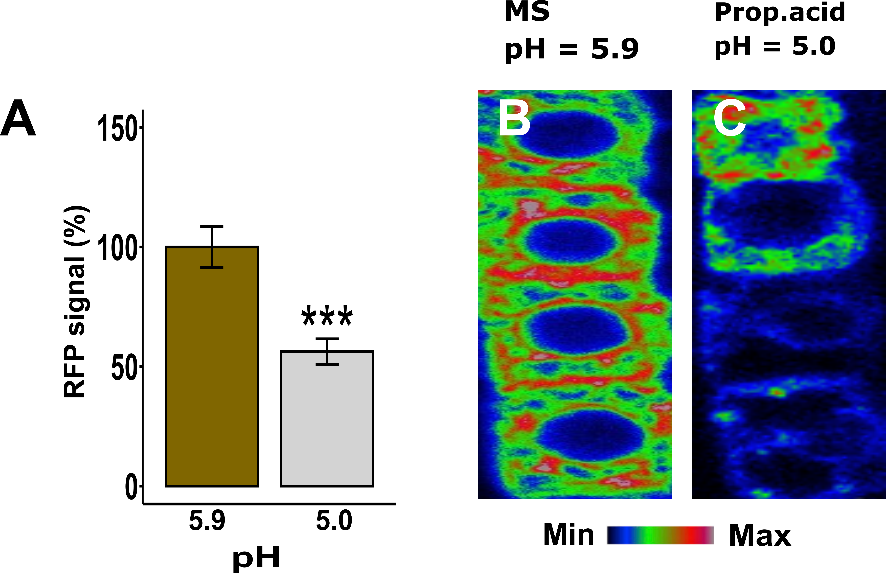


**Fig. S6.** The luminal HDEL-RFP is quenched with PM permeable propionic acid.

(A) Quantified RFP signal. The RFP signal in the MS (control) treatment is plotted as 100%. The asterisks indicate significant differences in comparison to the control MS treatment (*** P < 0.001, Student’s t-test). The significantly lower RFP signal after treatment with the propionic acid indicates that the luminal HDEL-RFP is quenched by the acidic pH. Error bars represent SEM from the total number of seedlings analysed from three biological experiments (n > 12 per single experiment). Scale bar = 10µm. (B-C) Images of color-coded root cells to better visualize the RFP intensity changes after the acidic treatment. Scale bar, 10 µm.


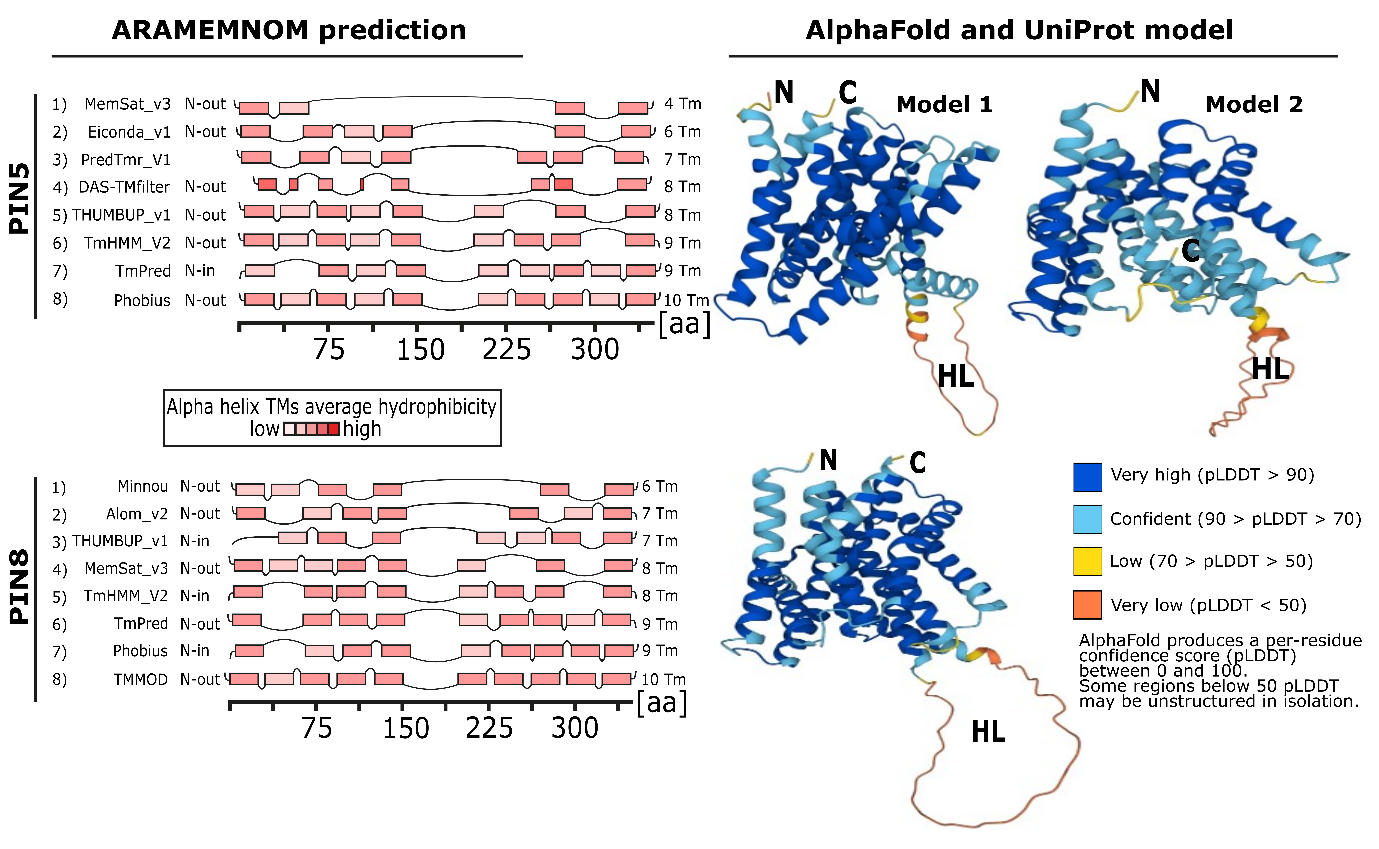


**Fig. S7.** Predicted topology of PIN5 and PIN8 proteins. The PIN5 and PIN8 predicted membrane topologies are obtained from the ARAMEMNOM, plant membrane protein database, and the 3D structures are retrieved from AlphaFold database on October 6, 2023. ARAMEMNOM predicts four to ten and six to ten TM helices of PIN5 and PIN8 respectively. AlphaFold predicted two different versions of *Arabidopsis thaliana* PIN5 gene models (model 1 identifier: AF-Q9FFD0-F1, model 2 identifier: AF-A0A1P8BEE7-F1) and a single model for PIN8 gene (AF-Q9LFP6-F1). The second model of PIN5 indicates that the C-terminal of the protein is ended in the TM helices the protein contains eight helices.


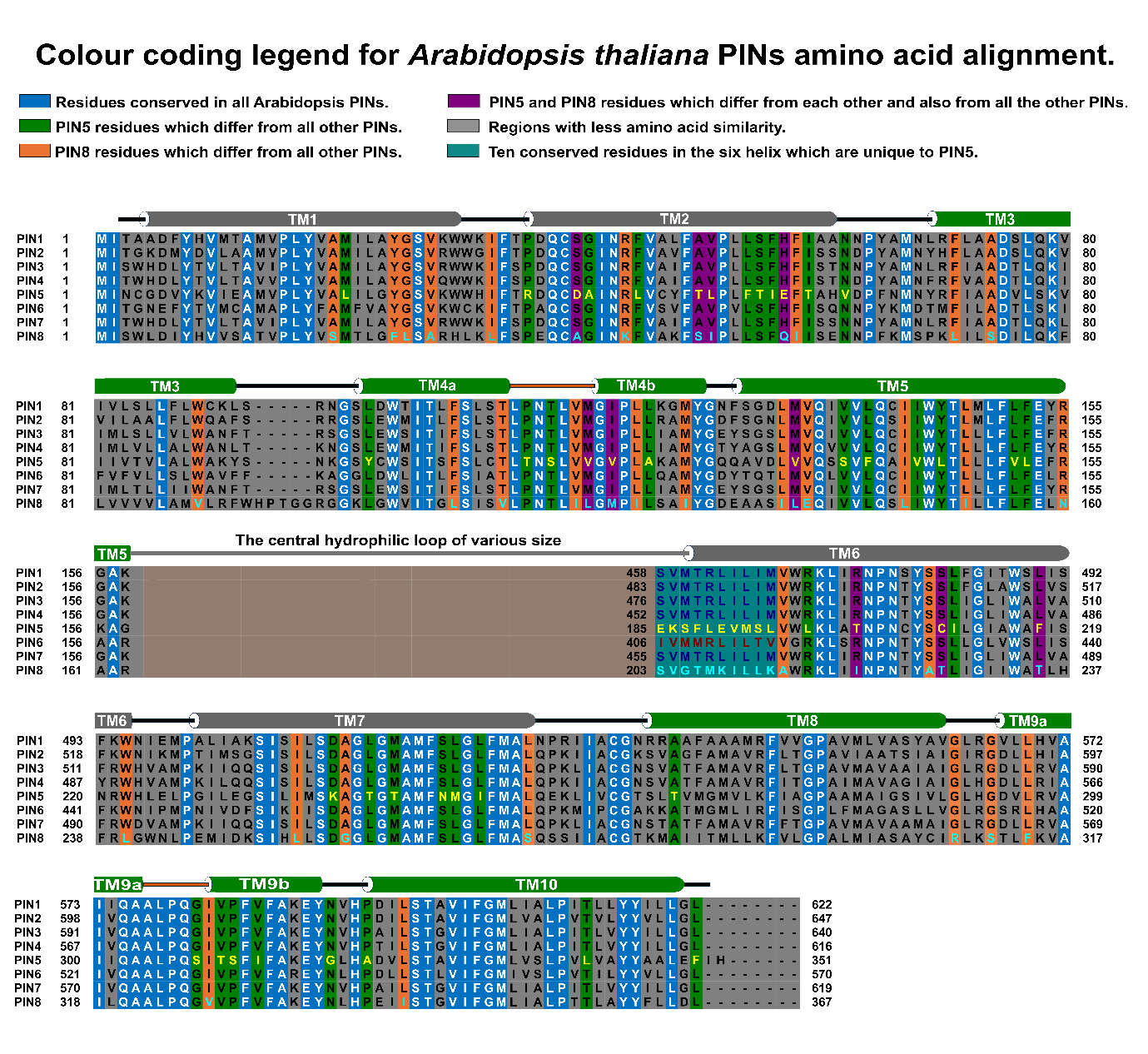


**Fig. S8.** Amino acid sequence alignment of *Arabidopsis thaliana* PINs’ transmembrane domains. To compare the differences within the highly conserved helices among the PINs, the sequence of various size and highly variable central hydrophilic loop was excluded from the analysis. The grey TM helices represent scaffold domain, and the green helices represent transport domain [36]. The PIN5 sequence contains various residues which are distinct from all the other PINs both in the scaffold and transporter domain. At the start of the six transmembrane helices all PINs contain ten conserved residues which are the same in the canonical PINs, but differ for the noncanonical PINs, and are unique in PIN5. Within the ten conserved amino acids, except for the PIN5, all PINs contain ''IL'' before the last two amino acids. The PIN sequences were acquired from UniProt under the following accession numbers: AtPIN1: Q9C6B8, AtPIN2: Q9LU77, AtPIN3: Q9S7Z8, AtPIN4: Q8RWZ6, AtPIN5: Q9FFD0, AtPIN6: Q9SQH6, AtPIN7: Q940Y5, AtPIN8: Q9LFP6.


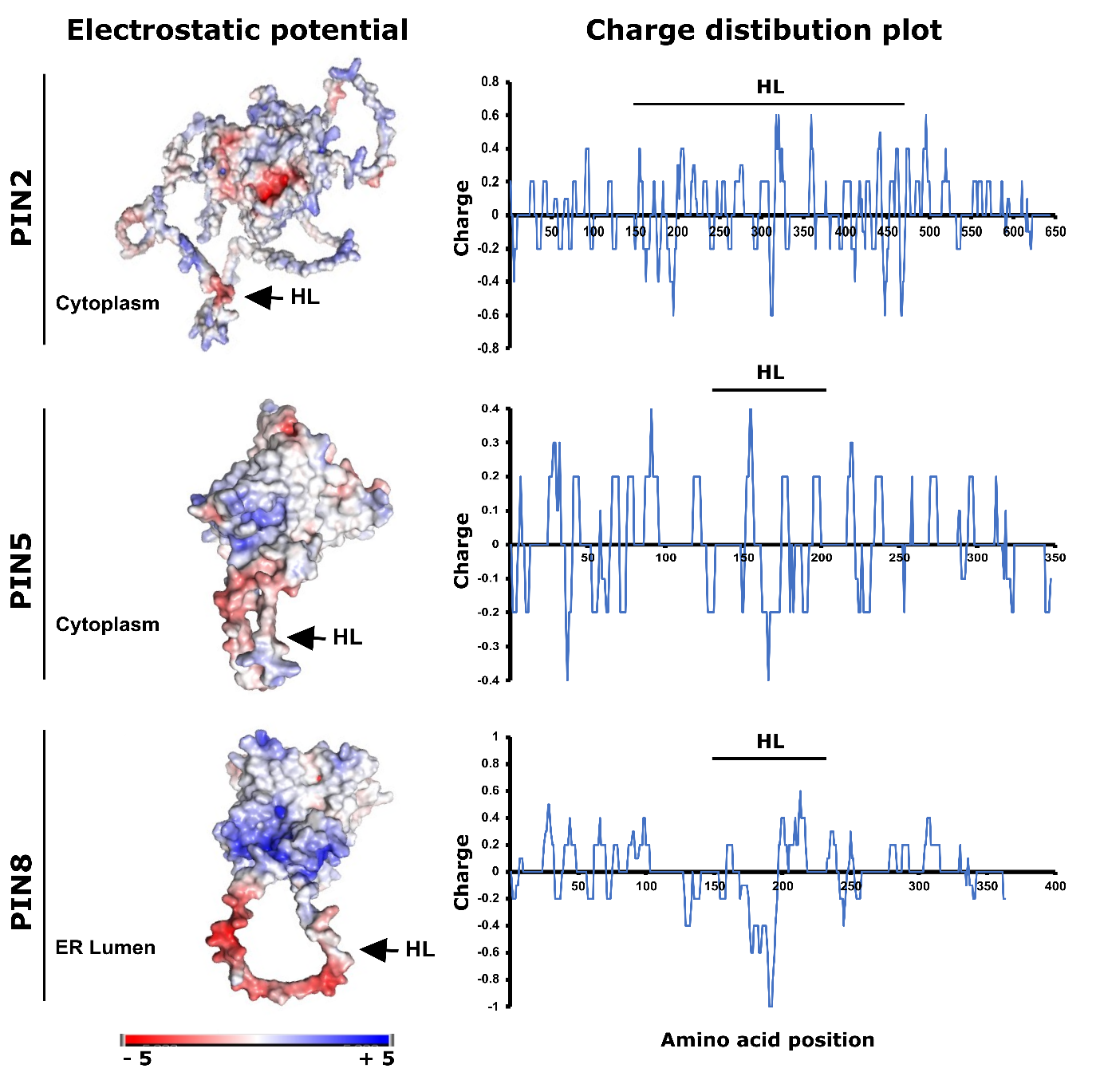


**Fig. S9.** Amino acid charge distribution in PIN5 and PIN8 indicated by electrostatic potential and charge distribution plot. The three-dimensional structure of the proteins was obtained from AlphaFold database, and the surface electrostatic potential was displayed using PyMol software. Positive potential is highlighted in blue and negative potential is indicated in red. The charge distribution plot was retrieved through EMBOSS webserver. The positively charged residues are distributed in accordance with the “positive inside rule” and consequently the hydrophilic loops of PIN2, as published, and PIN5 are positioned in the cytoplasm while the negatively charged amino acids in PIN8 HL are consistent with its non-cytoplasmic localization.

**Table S1.** The lists of primers used to generate the constructs.

|  | | | |
| --- | --- | --- | --- |
| Name of the construct or insert | | Primer name | Primer sequence |
| PIN2 promoter | | Pro_FP | GGGTGCAAGGATATCATTACCAGTACCG |
|  |  | Pro_RP | GGGTTTGATTTACTTTTTCCGGCGAGAG |
| N-terminal GFP fusion | GFP | eGFP-attB1-FP | AAAAAAGCAGGCTTCAACATGGTGAGCAAGG |
|  |  | eGFP-EcoRI-R | CGTCGCGAATTCCTTGTACAGCTCGTCC |
|  | PIN8:GFP | P8_EcoRI_FP | CATAGCGAATTCATGATCTCCTGGCTCGATATC |
|  |  | P8_attB2_RP | CAAGAAAGCTGGGTCTCATAGGTCCAATAGAAAATAATATGC |
|  | PIN5:GFP | P5_EcoRI_FP | GCACCGCTCGGAATTCATGATAAATTGTGGAGAT |
|  |  | P5_attB2_RP | CAAGAAAGCTGGGTCTCAATGAATAAACTCCAGAGCTGC |
| C-terminal GFP fusion | PIN5:GFP | attB1_P5_FP | GGGGACAAGTTTGTACAAAAAAGCAGGCTTCACCATGATAAATTGTGGAGAT |
|  |  | attB2_P5_RP | GGGG AC CAC TTT GTA CAA GAA AGCTGGGTCATGAATAAACTCCAGAGCTGC |
|  | PIN8:GFP | attB1_P8_FP | GGGGACAAGTTTGTACAAAAAAGCAGGCTTCAACATGATCTCCTGGCTCGATATC |
|  |  | attB2_P8_RP | GGGG AC CAC TTT GTA CAA GAA AGCTGGGTCTAGGTCCAATAGAAAATAATATGC |
| PIN5 GFP1 | Fragment 1 | attB1_P5_FP | AAAAAAGCAGGCTTCACCATGATAAATTGTGGAGAT |
|  |  | XbaI.P5_Fr1_RP | GCACGGAGAGCGTCTAGAGAGACGGTTTATAG |
|  | Fragment 2 | EcoRI.P5_Fr2_FP | CGGGGGATTATGGAATTCGTTTGCTATTTCACCCTG |
|  |  | P5_attB2_RP | CAAGAAAGCTGGGTCTCAATGAATAAACTCCAGAGCTGC |
| PIN8 GFP1 | Fragment 1 | attB1_P8_FP | AAAAAAGCAGGCTTCAACATGATCTCCTGGCTCGATATC |
|  |  | XbaI.P8_Fr1_RP | GCACGGAGAGCGTCTAGATGAGAAGAGCTTTAG |
|  | Fragment 2 | EcoRI.P8_Fr2_FP | GTCACCATCGAATTCCCCGAACAATGC |
|  |  | P8_attB2_RP | CAAGAAAGCTGGGTCTCATAGGTCCAATAGAAAATAATATGC |
| GFP | | eGFP-XbaI-F | TCGGAGTCTAGAATGGTGAGCAAGGGCG |
|  |  | eGFP-EcoRI-R | CGTCGCGAATTCCTTGTACAGCTCGTCC |
